# Wheat Heat Shock Factor TaHsfA6f Increases ABA Levels and Enhances Tolerance to Multiple Abiotic Stresses in Transgenic Plants

**DOI:** 10.3390/ijms21093121

**Published:** 2020-04-28

**Authors:** Huihui Bi, Yue Zhao, Huanhuan Li, Wenxuan Liu

**Affiliations:** National Key Laboratory of Wheat and Maize Crop Science, Henan Agricultural University, Zhengzhou 450002, China

**Keywords:** wheat, Hsf, gene expression, transgenic, *Arabidopsis*, RNA-seq

## Abstract

Abiotic stresses are major constraints limiting crop growth and production. Heat shock factors (Hsfs) play significant roles in mediating plant resistance to various environmental stresses, including heat, drought and salinity. In this study, we explored the biological functions and underlying mechanisms of wheat *TaHsfA6f* in plant tolerance to various abiotic stresses. Gene expression profiles showed that *TaHsfA6f* has relatively high expression levels in wheat leaves at the reproductive stage. Transcript levels of *TaHsfA6f* were substantially up-regulated by heat, dehydration, salinity, low temperature, and multiple phytohormones, but was not induced by brassinosteroids (BR). Subcellular localization analyses revealed that TaHsfA6f is localized to the nucleus. Overexpression of the *TaHsfA6f* gene in *Arabidopsis* results in improved tolerance to heat, drought and salt stresses, enhanced sensitivity to exogenous abscisic acid (ABA), and increased accumulation of ABA. Furthermore, RNA-sequencing data demonstrated that *TaHsfA6f* functions through up-regulation of a number of genes involved in ABA metabolism and signaling, and other stress-associated genes. Collectively, these results provide evidence that *TaHsfA6f* participates in the regulation of multiple abiotic stresses, and that *TaHsfA6f* could serve as a valuable gene for genetic modification of crop abiotic stress tolerance.

## 1. Introduction

Abiotic stresses, such as heat, drought, and salinity, have a tremendous impact on crop growth and production. Plant adaptation to abiotic stresses involves a series of sophisticated regulatory mechanisms. Plant heat shock factors (Hsfs) represent important players in the intricate regulatory network through modulating the expression of genes responsive to a variety of environmental stresses [1]. It was reported that Hsfs regulate the transcription of heat shock protein (*Hsp*) genes and other stress-inducible genes by recognizing palindromic heat shock elements (HSEs: 5′-AGAAnnTTCT-3′) within the promoter regions of these genes [2,3]. The protein products of *Hsp* genes function as molecular chaperones and assist in protein folding, assembly, and translocation, thus protecting plants from impairment under stress conditions [4]. To date, *Hsf* gene families have been identified and analyzed at the genome level in many plant species. For example, 21 *Hsf* genes were identified in *Arabidopsis* [5], 25 in rice [6], 25 in maize [7], and 38 in soybean [8].

Similar to other transcription factor families, the Hsf family has common structural features, including a highly conserved DNA-binding domain (DBD) and an oligomerization domain (HR-A/B regions) [5]. The N-terminal DBD allows Hsf proteins to recognize HSEs in target promoter regions and thus to regulate the expression of downstream genes. The HR-A/B domain enables Hsf proteins to form homologous trimers to facilitate binding to Hsp promoters. Moreover, some Hsf proteins also contain nuclear localization signals (NLSs) and a C-terminal activation domain characterized by short peptide motifs (AHA motifs). Based on the characteristics of the HR-A/B domains, plant Hsfs are generally grouped into three classes, HsfA, HsfB, and HsfC [5,9]. The Hsf family has been described in detail in model organism *Arabidopsis* [5,6]. *Arabidopsis* HsfA proteins function as positive regulators of heat stress-responsive genes, which results in the accumulation of HSPs and other proteins that increase thermotolerance [1]. HsfB may serve as coregulators or repressors of the HsfA genes, while the functions of HsfC remain unclear [10].

Thus far, studies about plant Hsfs have mainly focused on their roles in heat stress responses. For example, in *Arabidopsis*, *AtHsfA1a* and *AtHsfA1b* were shown to play important roles in the early phase of the heat stress response [5]; *AtHsfA1a/b* double-knockout mutants are considerably impaired in the early transient mRNA accumulation of *HSPs* [11]. AtHsfA2 was found to be an essential regulator of plant acquired thermotolerance in the recovery period of heat stress and is directly regulated by HsfA1 [12]. *AtHsfA3* transcript levels were induced by heat stress, and the overexpression of *AtHsfA3* showed remarkably elevated thermotolerance, while a *hsfA3* T-DNA insertion mutant had decreased thermotolerance [13]. More recently, *AtHsfA3* has been revealed to play important roles in the regulation of heat stress-induced oxidative stress signaling [14]. Additionally, *AtHsfA6b* was reported to be involved in ABA signaling and required for ABA-mediated heat stress resistance [15]. Several Hsfs in other plant species, including tomato *SlHsfA1* [16], tall fescue *FaHsfA2c* [17], wheat *TaHsfA6e* [18], and maize *ZmHsf05* [19], have also been proved to participate in thermotolerance.

In addition to heat stress, Hsfs have been demonstrated to play a role in other abiotic stress responses. *Arabidopsis HsfA1s* are involved in salt, osmotic, and oxidative stresses [20]. Both *HsfA1b* and *HsfA3* participate in drought stress responses [21], while *HsfA2* has been shown to confer improved tolerance to several abiotic stresses including salt, osmotic stress, and anoxia stress [12,22]. *AtHsfA6b* increased tolerance to salt and drought stresses when overexpressed in *Arabidopsis* [15]. Recently, it was found that the expression of *AtHsfA7b* is up-regulated by salt stress, and that AtHsfA7b protein directly binds to E-box-like motifs and/or HSEs to regulate a series of genes involved in salt tolerance [23].

The studies mentioned above have clearly indicated that Hsfs mediate cross-talk between heat stress and other abiotic stress responses. Previous studies have demonstrated that *TaHsfA6f* plays a role in increasing thermotolerance via regulation of heat stress protection genes in wheat [24]. In this study, we explored the biological functions and underlying mechanisms of wheat TaHsfA6f in plant tolerance to multiple abiotic stresses. To this end, we cloned the *TaHsfA6f* gene from the heat and drought tolerant wheat cv. TAM107. We analyzed the expression patterns of *TaHsfA6f* during multiple abiotic stresses and phytohormones, and investigated the functions of *TaHsfA6f* via heterologous overexpression and RNA-seq analyses. It was shown that *TaHsfA6f* is a positive regulator of multiple abiotic stresses in plants and it may be associated with the ABA signaling pathway. Our results revealed the potential value of *TaHsfA6f* in the genetic improvement of crop abiotic stress tolerance.

## 2. Results

### 2.1. Gene Expression of TaHsfA6f is Up-Regulated by Various Abiotic Stresses and Phytohormones

**Figure 1:**
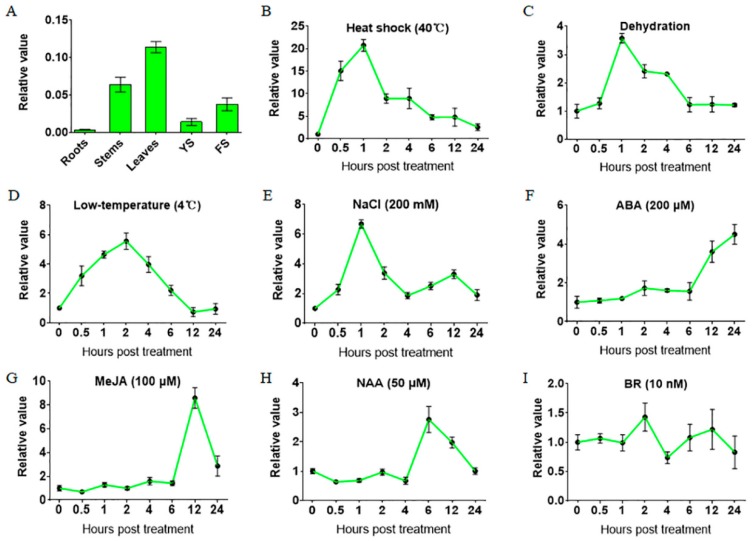
Gene expression patterns of *TaHsfA6f*. (**A**) Relative expression levels of *TaHsfA6f* in various wheat organs. Total RNA was isolated from roots, stems, leaves, young spikes (YS), and flowering spikes (FS). The data represent means of three biological replicates ± SD. (B-I) Gene expression patterns of *TaHsfA6f* during abiotic stresses and phytohormones. Total RNA was extracted from plants treated with 40 °C heat shock (**B**), dehydration (**C**), 4 °C low temperature (**D**), 200 mM NaCl (**E**), 200 µM ABA (**F**), 100 mM MeJA (**G**), 50 mM NAA (**H**), and 10 nM BR (**I**), for the indicated time points. The relative expression level was measured by qPCR and calculated by the comparative CT method. Error bars indicate standard deviations (SD).

The coding and genomic sequences of the *TaHsfA6f* gene were obtained by PCR amplification using the cDNA and genomic DNA of the leaves of wheat variety TAM107 as a template, respectively. A sequence alignment between the obtained coding sequence and genomic sequence revealed that the *TaHsfA6f* gene contains one intron and two exons (Figure S1A). To disclose the phylogenetic relationship of TaHsfA6f, the amino acid sequence of TaHsfA6f was blasted against the UniProt database, and the homologous sequences of TaHsfA6f in other species were obtained. Phylogenetic analyses of TaHsfA6f and the sequences from other species showed that TaHsfA6f and its homologous proteins diverged between monocotyledon and dicotyledon species (Figure S1B). Among the monocotyledon branches, TaHsfA6f had the highest similarity with the barley protein A0A287PMR3. It was also found that the homologous protein of TaHsfA6f in *Arabidopsis thaliana* is AtHsfA6b (Q9LUH8) (Figure S1B). In order to examine the expression patterns of the *TaHsfA6f* gene in wheat tissues, we selected five tissues (root, stem, leaf, young spikes and flowering spikes) at the reproductive stage of Chinese spring, and analyzed the gene expression by qPCR. It was found that *TaHsfA6f* gene was expressed in all detected tissues, with the highest expression level in leaves and the lowest in roots under normal growth conditions (Figure 1A).

Next, we investigated the expression patterns of the *TaHsfA6f* gene in response to different abiotic stresses and phytohormones. As shown in Figure 1, the *TaHsfA6f* gene was greatly and quickly (within an hour) up-regulated by heat shock, drought, low temperature, and NaCl treatments. Remarkably, the gene expression of *TaHsfA6f* was up-regulated by 40 °C heat stress, the highest level being 20-fold greater than the untreated control (0 h), and peak values appearing 1 h after heat stress treatment (Figure 1B). By contrast, the responses of *TaHsfA6f* to phytohormones were slow. Specifically, the expression of the *TaHsfA6f* gene was significantly increased at 12 h by both ABA and MeJA, and at 6 h by NAA (Figure 1F–H). No obvious induction of *TaHsfA6f* gene expression was observed for BR (Figure 1I).

### 2.2. Subcellular Location of TaHsfA6f Protein

**Figure 2:**
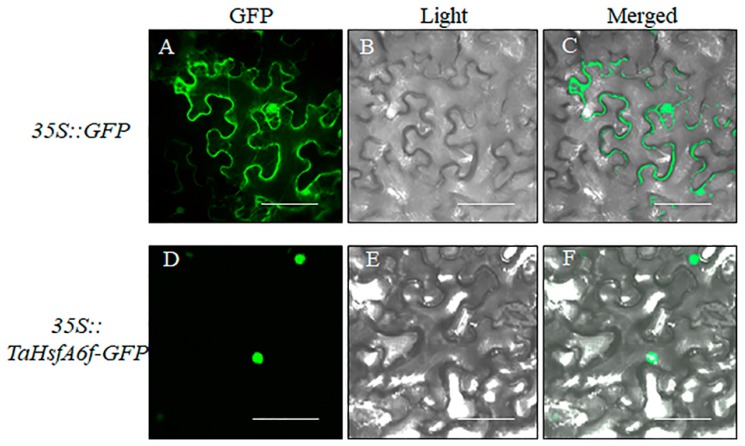
Subcellular localization of TaHsfA6f protein by *Nicotiana benthamiana* leaf epidermal cells transient expression. *35S::GFP* and *35S::TaHsfA6f-GFP* plasmids were transformed into *Nicotiana benthamiana* leaf epidermal cells and signals were visualized with laser confocal-scanning fluorescence microscopy. From left to right, the photographs were taken in the dark-field for green fluorescence (GFP) (**A**,**D**), under bright-field for the morphology of the cell (Light) (**B**,**E**), and overlays of the GFP signals and bright-field (Merged) (**C**,**F**). The scale bars represent 40 μm.

Subcellular localization of TaHsfA6f was performed in *Nicotiana benthamiana* leaf epidermal cells transformed with constructs containing *35S::TaHsfA6f-GFP* and *35S::GFP*, respectively. As shown in Figure 2, the green fluorescence of cells with GFP was seen in membrane, nucleus and cytosol, while it was only present in the nucleus of cells with TaHsfA6f-GFP, indicating that TaHsfA6f is a nucleus protein.

### 2.3. Overexpression of TaHsfA6f Confers Improved Tolerance to Heat, Drought and Salt Stresses in Arabidopsis

**Figure 3:**
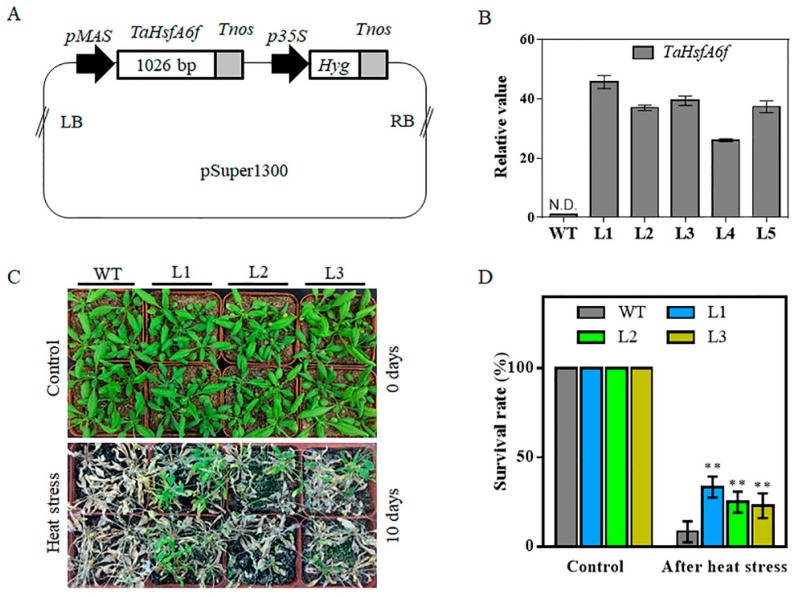
Thermotolerance of WT and transgenic *Arabidopsis* plants. (**A**) Schematic representation of the construct used for *Arabidopsis* transformation. LB left border, *pMAS* mannopine synthase promoter, *Tnos* nopaline synthase gene (NOS) terminator, *Hyg* Hygromycin-resistance gene, RB right border. (**B**) Relative expression levels of *TaHsfA6f* in the leaves of T3 transgenic *Arabidopsis* lines via qPCR analysis. Each value is the mean of triplicate experiments. Bars indicate standard deviations. WT wild-type, N.D = Not Detected. (**C**) Comparison of thermotolerance among WT and three transgenic lines (L1, L2, and L3). (**D**) Survival rates of WT and three transgenic lines after heat stress at 40/32 °C (day/night) for 10 days. For each experiment, 20 plants per line were used; values are means ± SD from three independent measurements (** *p* < 0.01 by Student’s *t*-test).

In order to reveal the biological functions of wheat *TaHsfA6f* gene, we constructed an overexpression vector (*pMAS::TaHsfA6f*) of *TaHsfA6f* (Figure 3A), which was transferred into *Agrobacterium* GV3101, and the positive *Agrobacterium* strain with *TaHsfA6f* was then transformed into *Arabidopsis*. Five single copy transgenic *Arabidopsis* lines were obtained after hygromycin B screening. The expression of *TaHsfA6f* in transgenic *Arabidopsis* was detected in all *pMAS::TaHsfA6f* transgenic lines but not in the WT plants (Figure 3B). Three independent T3 transgenic lines (L1, L2 and L3) exhibiting the highest *TaHsfA6f* expression levels were selected for further analyses.

Firstly, we examined whether *TaHsfA6f* overexpression could improve tolerance to heat stress in transgenic *Arabidopsis* plants. As shown in Figure 3C,D, under normal conditions (control), no differences were found between transgenic and WT plants and all of them grew well. Under a heat stress of 40/32 °C (day/night) for 10 days, however, the survival rates of transgenic *Arabidopsis* L1 (33%), L2 (25%) and L3 (23%) were clearly higher than those of WT (8%), indicating that overexpression of the *TaHsfA6f* gene could improve the heat tolerance of transgenic *Arabidopsis*.

**Figure 4:**
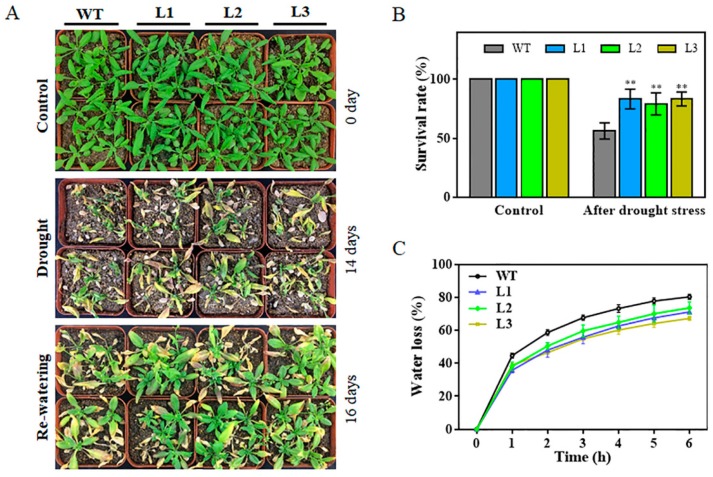
Comparison of drought tolerance among WT and three *TaHsfA6f* overexpressing lines. (**A**) Drought-tolerant phenotypes and (**B**) survival rates of WT and transgenic lines. Water was withheld for 14 days; afterwards, re-watering was applied and survival rates were calculated two days after re-watering. (**C**) Water loss rates of WT and transgenic lines. The data presented are means ± SD of three replicates (** *p* < 0.01 by Student’s *t*-test).

To investigate whether *TaHsfA6f* overexpression could enhance drought stress tolerance in plants, three-week-old transgenic and WT plants were subjected to drought stress by withholding watering for 14 days, and then a two-day growth recovery was conducted. The three transgenic lines (L1, L2, and L3) exhibited better growth status than WT (Figure 4A) and the survival rates of the transgenic lines were significantly higher than that of the WT (Figure 4B). Water loss rates serve as an important indicator of drought tolerance in plants. Water loss rates of detached leaves were recorded every hour at room temperature for both the WT and the three transgenic lines. As shown in Figure 4C, the transgenic lines exhibited significantly lower water loss rates compared to the WT.

**Figure 5:**
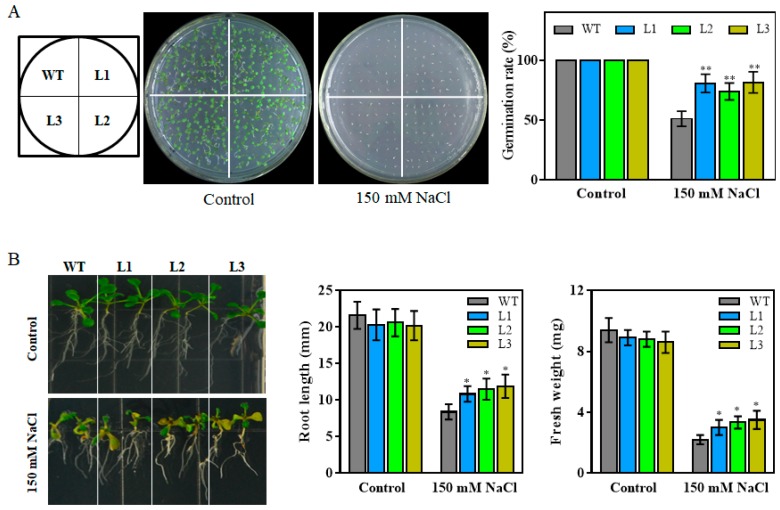
Effect of salt stress on WT and transgenic *Arabidopsis* plants. (**A**) Comparison of germination rates among WT and three transgenic lines (L1, L2, and L3) under salt stress (NaCl). Seeds were germinated on 1/2MS plates supplemented with 0 (control) or 150 mM NaCl. The data was recorded after four days of germination. (**B**) Growth and biomass accumulation of seven-day-old seedlings of WT and three transgenic lines treated with 150 mM NaCl for five days. Values are means ± SD from three independent measurements (* p < 0.05, ** *p* < 0.01 by Student’s *t*-test).

To reveal a potential role of *TaHsfA6f* in salt stress responses, 150 mM NaCl was applied to media with seeds or seven-day-old seedlings of transgenic and WT plants. As shown in Figure 5A,B, there was no difference between WT and transgenic plants in seed germination rates, root length, and fresh weight under control conditions. However, when grown on 150 mM NaCl-containing 1/2MS media, all transgenic plants displayed increased seed germination rates, root length, and fresh weight compared with WT. These results indicated that *TaHsfA6f* overexpression in transgenic *Arabidopsis* plants enhances their tolerance to heat, drought and salt stresses.

### 2.4. Transgenic Arabidopsis Plants are Hypersensitive to ABA

**Figure 6:**
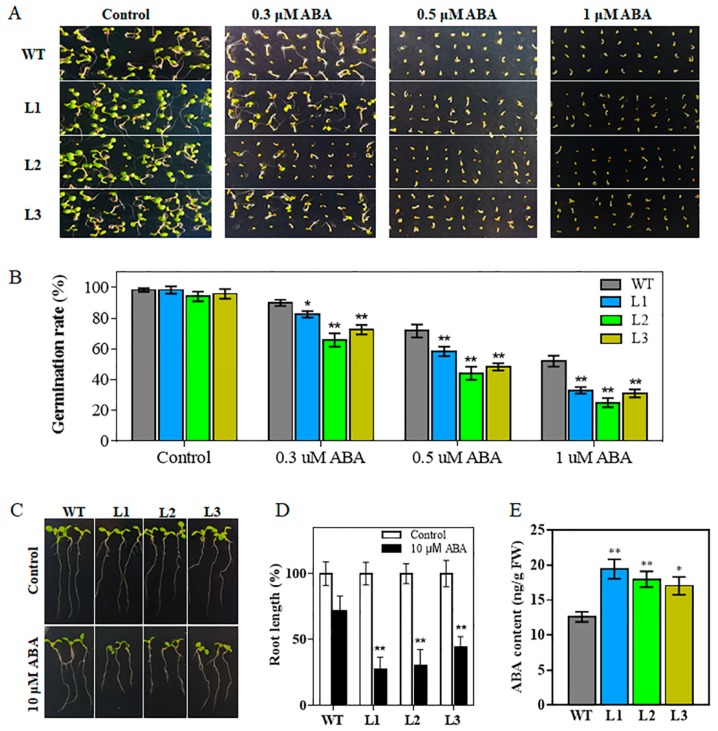
Effect of ABA on *TaHsfA6f* transgenic *Arabidopsis* plants. (**A**–**B**) Germination assays of *TaHsfA6f* transgenic plants under ABA treatment. Seed germination ratios of WT and three *TaHsfA6f* transgenic lines on 1/2MS agar media with different concentrations of ABA. (**C**) Morphology of WT and three *TaHsfA6f* transgenic seedlings grown vertically on 1/2MS agar media with or without 10-μM ABA. The photographs were taken three days after seedlings were transferred to the indicated agar media. (**D**) Root length recorded before photographing. (**E**) Levels of endogenous ABA in the leaves of WT and the three transgenic lines under normal conditions. Data shown are means ± SD of three independent experiments (* p < 0.05, ** *p* < 0.01 by Student’s *t*-test).

Since the expression of *TaHsfA6f* was induced by ABA treatment (Figure 1F), we investigated whether *TaHsfA6f* plays a role in ABA signaling pathway. As shown in Figure 6A,B, there was no noticeable difference in seed germination between transgenic lines (L1, L2, and L3) and WT on ABA-free media (Control). However, on media containing 0.3-, 0.5- or 1-μM ABA, the germination capability of seeds from the transgenic lines was much more reduced than that of the WT seeds (Figure 6A,B). In terms of root elongation, no significant growth difference was observed between transgenic and WT seedlings under control conditions (Figure 6C,D). However, on 10-μM ABA, the root elongation of the transgenic seedlings was reduced compared with that of the WT seedlings (Figure 6C,D). These results showed that transgenic plants are hypersensitive to ABA during seed germination and seedling growth.

### 2.5. Overexpression of TaHsfA6f Increases Cellular ABA Content

As shown above, overexpression of the *TaHsfA6f* gene in *Arabidopsis* increased heat, drought and salt tolerance. ABA plays a pivotal role in plants’ resistance to adverse environments. To study whether *TaHsfA6f* affects the biosynthesis of ABA to improve abiotic stress tolerance, we measured the content of endogenous ABA using a UPLC–MS/MS system. The content of ABA was significantly higher in transgenic plants (L1, L2, and L3) than that of the WT plants under normal growth conditions (Figure 6E). These results clearly revealed that overexpression of the *TaHsfA6f* gene enhances cellular ABA levels.

### 2.6. Transcriptome Analysis of TaHsfA6f Transgenic Arabidopsis

**Figure 7:**
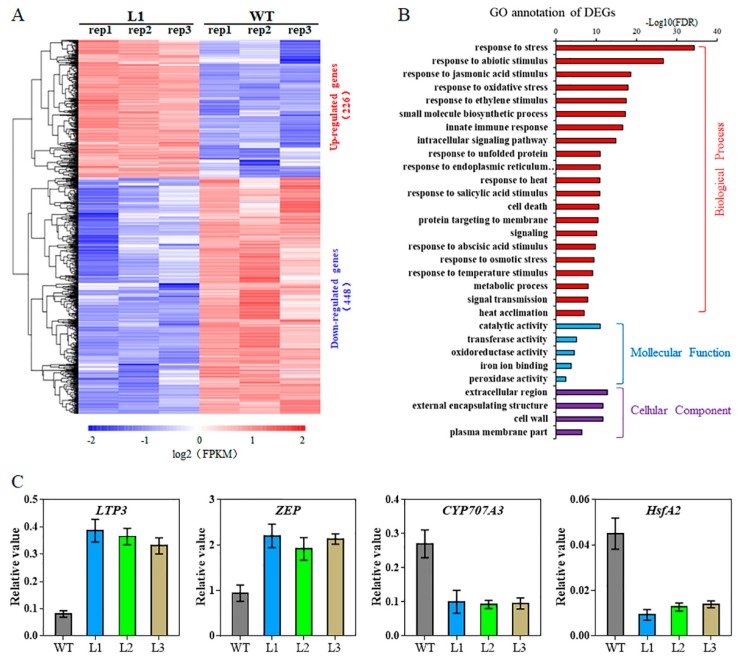
Transcriptomic analyses of *TaHsfA6f* transgenic *Arabidopsis*. (**A**) Heat map of DEGs in leaves of transgenic vs. WT plants. The heat map represents the gene expression levels of 674 significant DEGs (log2Foldchange > 1 and FDR < 0.001) between transgenic (L1: rep1, rep2 and rep3) and WT (WT: rep1, rep2 and rep3) plants. The indicated scale is the log2 value of the normalized level of gene expression. (**B**) Gene ontology classification for DEGs in *TaHsfA6f* transgenic and WT plants. (**C**) qPCR validation of RNA-seq results for DEGs. Error bars indicate standard deviations.

To decipher the possible molecular mechanisms of *TaHsfA6f* functioning in plant stress responses, genes with altered expression levels were identified in the transgenic plants using the RNA-seq approach. A total of 226 and 448 genes were found to be up- and down-regulated (fold change > 2, FDR < 0.001), respectively, in the transgenic plants compared with WT (Figure 7A and Table S2). Gene Ontology (GO) analyses revealed that the DEGs were highly enriched in the entries of ‘response to stress’ and ‘response to abiotic stimulus’ in the biological process category (Figure 7B). In the molecular function category, DEGs were highly accumulated in ‘catalytic activity’, oxidoreductase activity, and antioxidant activity (Figure 7B). Based on gene annotation, we found that many up-regulated DEGs in the transgenic plants are Hsp genes, and most of them were small Hsp genes (Table 1). In addition, other stress-responsive genes, such as three galactinol synthase genes (*GolS1*, *GolS2*, *GolS4*), two lipid-transfer protein-encoding genes (*LTP3* and *LTP4*), dehydration-responsive element-binding protein 1 genes (*DREB1A* and *DREB1C*), *ascorbate peroxidase 2* (*APX2*), and *late embryogenesis abundant group3* (*LEA3*) were up-regulated in the transgenic plants. Interestingly, *ZEP*, which encodes an enzyme important in ABA biosynthesis, was found to be slightly up-regulated in transgenic plants. *CYP707A3* (encoding a protein involved in ABA catabolism) and *HsfA2* were found to be down-regulated in transgenic plants. Four genes from Table 1 were randomly selected and further validated using qPCR. For all four genes, the fold changes in gene expression determined by qPCR agreed well with the RNA-seq data (Figure 7C).

## 3. Discussion

Wheat (*Triticum aestivum* L.), belonging to the Poaceae family, is one of the world’s most important staple foods. Wheat production is challenged by various stresses, such as high temperature, drought and salinity. Therefore, it is of great significance to characterize wheat stress response genes for their modification to enhance crop adaptability. A previous study has shown that the wheat *TaHsfA6f* gene was induced in response to heat stress; *TaHsfA6f* overexpression transgenic plants showed improved thermotolerance [24]. In this study, we found that *TaHsfA6f* was quickly (within an hour) and dramatically up-regulated by heat stress (Figure 1B) and overexpression of the *TaHsfA6f* gene improved the heat tolerance of transgenic *Arabidopsis* (Figure 3C,D). These results imply that the improvement of heat resistance by *TaHsfA6f* is conserved between monocot and dicot plant species. More importantly, we found that *TaHsfA6f* was up-regulated by various abiotic stresses (Figure 1), suggesting a potential role of *TaHsfA6f* in various stress responses. Indeed, *TaHsfA6f* transgenic *Arabidopsis* plants exhibited enhanced tolerance to drought and salt stresses (Figure 4 and Figure 5). Numerous studies have shown that plant hormones play important roles in plants adaptation to a variety of environmental stresses [25]. In the present study, we found that *TaHsfA6f* responds to ABA, MeJA, and NAA treatments (Figure 1). These results indicated that *TaHsfA6f* probably participates in a complex regulatory network in plant stress responses.

To explore the molecular mechanism underlying the involvement of *TaHsfA6f* in plant stress responses, we analyzed differential gene expression between transgenic *Arabidopsis* and WT plants through RNA-seq. A total of 674 DEGs were found in transgenic *Arabidopsis* compared to WT plants (Figure 7). As expected, the expression of several Hsp genes, including *Hsp22.0*, *Hsp25.3*, *Hsp26.5*, *Hsp18.1*, *Hsp17.6C*, *Hsp23.6*, *Hsp17.6*, *Hsp17.6B*, and *Hsp70-5/8* were increased markedly in transgenic plants (Table 1). It was reported that the Hsps function as molecular chaperones to prevent non-native aggregation and facilitate appropriate refolding of heat-damaged proteins [4]. *Arabidopsis GolS1*, *GolS2*, *GolS4* genes encoding for galactinol synthase, which are key enzymes of raffinose oligosaccharide synthesis, were also up-regulated in transgenic plants (Table 1). Those genes were previously reported to mainly function in drought, high-salinity, and osmotic stress tolerance [26,27,28,29]. Other stress-responsive genes, such as *APX2*, *LTP3*, *LTP4*, and *LEA3*, were also up-regulated in *TaHsfA6f* overexpressing plants. *Arabidopsis APX2*, encoding cytosolic ascorbate peroxidase, plays an important role in the acquisition of thermotolerance [30]. The expression of *APX2* contributes to maintaining the activity of the antioxidant system and protects plants against oxidative damage caused by adverse stresses [27,30]. *Arabidopsis LTP3* and *LTP4* were demonstrated to participate in the transportation of cuticular waxes, which protect plants against biotic and abiotic stresses; overexpression of *LTP3* increased drought and freezing tolerance in transgenic *Arabidopsis* [31]. In addition, overexpression of *AtLEA3* enhanced drought tolerance in *Arabidopsis* [32]. Our results agreed well with previous reports on other HsfAs. It was shown that overexpression of the *AtHsfA1*, *AtHsfA2*, and *AtHsfA3* activated the expression of a group of *Hsps* and stress-associated genes, which consequently led to improved abiotic stress tolerance in *Arabidopsis* [14,20,33].

Intriguingly, the expression of *Arabidopsis HsfA2*, which is a key regulator in abiotic stress responses, was significantly repressed in *TaHsfA6f* transgenic plants (Table 1). Previous studies have indicated that Hsfs could form homo- or heterotrimers, which then act on their own promoters and/or promoters of other HSF genes, increasing or decreasing the expression of these genes [34]. In this study, phylogenetic analyses showed that *Arabidopsis* AtHsfA6b is the closest member to TaHsfA6f (Figure S1B). AtHsfA6b cannot interact with itself, but interacts with the AtHsfA2 in the nucleus [15]. Therefore, we speculated that the repressed expression of *AtHsfA2* in *TaHsfA6f* transgenic plants might be due to the phenomenon that TaHsfA6f interacts with AtHsfA2 to form hetero-oligomers, suppressing *AtHsfA2* transcription. Future molecular biological evidence is required.

It is well known that ABA plays a crucial role in abiotic stress tolerance [25]. Several studies have indicated that Hsfs could connect ABA signaling. For instance, HsfA9 functions downstream of ABI3, a transcription factor in the ABA signaling pathway, and contributes to thermotolerance in *Arabidopsis* [35]. *AtHsfA6b* was induced by ABA and involved in the ABA signaling pathway and ABA-mediated thermotolerance and drought tolerance [15]. In this study, the expression of *TaHsfA6f* was induced by ABA treatment (Figure 1F). Overexpression of *TaHsfA6f* in *Arabidopsis* enhanced sensitivity to ABA during seed germination and seedling development (Figure 6). Additionally, we observed that the ABA levels in transgenic lines were increased (Figure 6E). RNA-seq analyses showed that the expression of *CYP707A3* was significantly down-regulated in the *TaHsfA6f* transgenic plants (Table 1). It was reported that *CYP707A3* is involved in ABA catabolism and functions as a determinant of endogenous ABA levels during dehydration–rehydration responses [36]. Moreover, the expression of *AtZEP* and *APX2* were up-regulated in the *TaHsfA6f* transgenic plants (Table 1). Overexpression of *AtZEP*, an enzyme important in ABA biosynthesis, improved drought and salt tolerance and caused an increase in endogenous ABA content [37]. *Arabidopsis* with constitutively high expression of *APX2* showed high ABA levels [38]. In addition, ABA can induce the expression of *GolS* genes, which contribute to oxidative stress tolerance by acting downstream of ABA signaling [39]. All of these findings suggest the possible involvement of *TaHsfA6f* in the ABA signaling pathway, and that the improved tolerance to heat, drought, and salt is at least partly attributed to ABA.

In conclusion, our results showed that overexpression of *TaHsfA6f* altered the expression levels of genes involved in the ABA metabolism and signaling, and other stress-related genes, thereby conferring heat, drought, and salt tolerance in *Arabidopsis*. It will be interesting to study the potential regulatory network of *TaHsfA6f* in ABA metabolism and/or ABA signaling pathways.

## 4. Materials and Methods

### 4.1. Plant Materials and Growth Conditions

Heat- and drought-tolerant wheat, cv. TAM107, was used for gene cloning and expression analyses. The seeds were grown hydroponically in a growth chamber at 22 °C with a 16-h photoperiod. For salt, and exogenous hormone treatments, 10-day-old wheat seedlings were transferred to growth media supplemented with 200-mM NaCl, 200-µM abscisic acid (ABA), 100-mM methyl jasmonate (MeJA), 50-mM a-naphthaleneacetic acid (NAA), or 10-nM brassinosteroids (BR), respectively. For the dehydration treatment, 10-day-old wheat seedlings were placed on filter papers at room temperature. Cold treatments were conducted by transferring 10-day-old wheat seedlings to a pre-cooled medium and placed in a growth chamber at 4 °C. Heat stress was carried out by placing plants in a 40 °C chamber. Shoots were collected separately at 0, 0.5, 1, 2, 4, 6, 12 and 24 h after each treatment. To study the tissue-specific expression of the *TaHsfA6f*, roots, stems, leaves, young spikes, and flowering spikes at the reproductive stage were collected from Chinese Spring wheat. All of the samples were immediately frozen in liquid nitrogen and stored at −80 °C for RNA extraction.

The *Arabidopsis* ecotype Columbia-0 was used as the wild-type. Seeds were surface sterilized with a 5% NaClO solution, washed five times with sterile water, and stratified at 4 °C for two days in the dark. The sterilized seeds were plated on Murashige and Skoog (MS) media containing 0.8% (*w/v*) agar and then transferred to a growth chamber. Growth conditions were 22 °C under a 16-h photoperiod, with a light intensity of 100 μmol m^−2^ s^−1^. After seven days, uniform seedlings were transplanted into soil and grown in the growth chamber.

### 4.2. Gene Cloning and Sequence Analysis

The *TaHsfA6f* sequence was retrieved from the IWGSC database (http://www.wheatgenome.org/) and used to design primers. The PCR for amplifying *TaHsfA6f* was conducted with the following program using high-fidelity Primestar polymerase (TaKaRa, Dalian, China): initial denaturation at 98 °C for 3 min; followed by 98 °C for 15 s, 60 °C for 20 s, 72 °C for 1 min with 34 cycles; and then 3 min at 72 °C. The PCR products were purified and cloned into the pEASY-Blunt cloning vector (TransGen, Beijing, China) and subjected to sequencing. Primers were designed using DNAMAN software. BLAST (https://www.uniprot.org/blast/) was used to search for the homologous sequences of TaHsfA6f in the UniProt databases. The phylogenetic tree was constructed with MEGA 7 software using the neighbor-joining (NJ) method [40]. All of the primers used are listed in Supplementary Table S1.

### 4.3. RNA Extraction and qPCR Analysis

The total RNA was isolated using TRIzol reagent (Invitrogen, Carlsbad, CA, USA) according to the manufacturer’s protocol. First-strand cDNA was generated using a Reverse Transcription System (Vazyme, Nanjing, China) following the manufacturer’s instructions. A real-time quantitative PCR (qPCR) experiment was performed using the SYBR Green PCR Master Mix kit (Tiangen Biotech, Beijing, China) and the IQ5 light cycler system (Bio-Rad, Hercules, CA, USA). The parameters for qPCR have previously been described [41]. The wheat *β-actin* and *AtActin2* genes were used as internal controls in wheat and *Arabidopsis*, respectively. The samples were assessed using three biological replicates. The relative gene expression was calculated using the comparative CT method [42].

### 4.4. Subcellular Localization Analysis

The coding sequence of the *TaHsfA6f* gene without the terminal codon was introduced in frame to the 5′ end of the *green fluorescent protein* (*GFP*) gene sequence in pCAM35s vector. The resulting construct was transformed into the *Agrobacterium* GV3101, which was used for transient expression in leaf epidermal cells of *Nicotiana benthamiana*. After *Agrobacterium* infiltration, plants were allowed to grow for two days at 22 °C with a 16-h-light/8-h-dark photoperiod before visualization under an Olympus FV10-ASW confocal laser scanning microscope.

### 4.5. Arabidopsis Transformation and Stress Treatments

The coding sequence of *TaHsfA6f* amplified by PCR from *pEASY-Blunt::TaHsfA6f* plasmid was cloned into the pSuper1300 vector harboring the *mannopine synthase* (*MAS*) promoter. The recombinant plasmid was transformed into the *Agrobacterium* GV3101, which was then used for transformation of wild type (Col-0) *Arabidopsis* using the floral-dip method. Transgenic plant seeds were screened on MS media containing 50 mg L^−1^ hygromycin. Putative positive seedlings were confirmed by qPCR.

For stress treatment experiments, three T3 transgenic lines and WT plants were germinated on MS agar plates for seven days. The healthy uniform seedlings were then transferred to 1/2MS media containing either 0- or 150-mM NaCl and planted vertically for five days, after which the fresh weight and root length of the plants were measured. Additionally, seven-day-old seedlings were transplanted into plastic pots containing the mixed soil of sand, peat, and perlite (1:1:1, *v/v/v*) and grown in a growth chamber for 14 days under normal growth conditions. The three-week-old plants were used for both heat and drought treatments. For heat stress, transgenic lines and WT plants were grown at 40/32 °C (day/night) with irrigation every day. The treatment lasted for 10 days and then survival rates were measured. For drought stress, water was withheld for 14 days. Afterwards, re-watering was applied, and survival rates were calculated two days after re-watering. Water loss rates were measured according to the method described by Zhao et al. [43]. The above experiments were repeated three times.

For the seed germination assay under stress treatments, the seeds of WT and three transgenic lines were sterilized and germinated on 1/2MS agar plates containing either 150-mM NaCl or ABA at different concentrations (0.3, 0.5 or 1 μM) following the method described previously [41]. Root elongation rate assay was carried out on 1/2MS agar media supplemented with 0- or 10-μM ABA according to Ling’s method [44].

### 4.6. Quantification of ABA Levels in Arabidopsis

The ABA content was measured using a method described previously [45]. ABA was extracted from flesh rosette leaves of three-week-old WT and transgenic *Arabidopsis* under normal growth conditions. Three biological replicates were used.

### 4.7. RNA-seq Analysis

Total RNA was extracted using Trizol reagent (TransGen, Beijing, China) from the leaves of three-week-old *Arabidopsis* seedlings of a transgenic line (L1) and WT plants under normal growth conditions. cDNA synthesis, libraries construction, sequencing, and raw reads analysis were conducted according to Zhao et al. [43]. The resulting libraries were sequenced on an Illumina HiSeq X Ten platform (Illumina, San Diego, CA, USA), resulting in 150 bp single-end reads. Three biological replicates were used. Approximately 4 GB of clean data were produced for each sample. The clean reads generated were aligned against the reference *Arabidopsis* genome using HISAT2 [46]. The number of sequencing reads generated from each sample was converted into FPKM (Fragments per Kilobase per Million Mapped Fragments) [47]. Differentially expressed genes (DEGs) were identified using the R package DEGseq [48]. The threshold value was set to |log2ratio| > 1 and false discovery rate (FDR) < 0.001. Gene Ontology (GO) analyses were performed using agriGO (a Web-based tool and database for gene ontology analysis; http://bioinfo.cau.edu.cn/agriGO/) [49]. RNA-Seq data reported here can be accessed with accession number PRJNA622833 in the Sequence Read Archive (SRA) at the National Center for Biotechnology Information (NCBI).

### 4.8. Statistical Analyses

Statistical analyses were performed in Microsoft Excel 2016. Data are represented as means ± standard deviation (SD). Differences between WT and transgenic lines were evaluated using a Student’s *t*-test. A single asterisk (*) represents a significant difference at *p* < 0.05, and a double asterisk (**) represents a significant difference at *p* < 0.01.

## Figures and Tables

**Table 1:** The list of DEGs in *TaHsfA6f* transgenic *Arabidopsis* that are highlighted in this paper.

Gene ID	Gene Symbol	Fold Change	Profile	Description
AT4G10250	HSP22.0	673.8	up	22.0 kDa heat shock protein
AT1G60470	GOLS4	571.1	up	Galactinol synthase 4
AT4G27670	HSP25.3	475.1	up	25.3 kDa heat shock protein
AT3G09640	APX2	268.1	up	L-ascorbate peroxidase 2
AT1G52560	HSP26.5	149.4	up	26.5 kDa heat shock protein
AT5G59720	HSP18.1	84.1	up	18.1 kDa class I heat shock protein
AT1G53540	HSP17.6C	46.1	up	17.6 kDa class I heat shock protein 3
AT4G25200	HSP23.6	31.5	up	23.6 kDa heat shock protein
AT5G59310	LTP4	19.0	up	Non-specific lipid-transfer protein 4
AT5G12020	HSP17.6	11.9	up	17.6 kDa class II heat shock protein
AT4G21320	HSA32	9.7	up	HEAT-STRESS-ASSOCIATED 32
AT5G12030	HSP17.7	9.0	up	17.7 kDa class II heat shock protein
AT2G47180	GOLS1	8.8	up	Galactinol synthase 1
AT2G29500	HSP17.6B	6.5	up	17.6 kDa class I heat shock protein 2
AT1G02820	LEA3	4.5	up	late embryogenesis abundant 3
AT5G59320	LTP3	4.4	up	Non-specific lipid-transfer protein 3
AT5G37670	HSP15.7	4.3	up	15.7 kDa heat shock protein
AT1G16030	HSP70-5	3.0	up	Heat shock 70 kDa protein 5
AT2G32120	HSP70-8	3.0	up	Heat shock 70 kDa protein 8
AT1G56600	GOLS2	2.6	up	Galactinol synthase 2
AT4G25480	DREB1A	2.2	up	Dehydration-responsive element-binding protein 1A
AT4G25470	DREB1C	2.2	up	Dehydration-responsive element-binding protein 1C
AT5G66400	RAB18	2.1	up	Dehydrin Rab18
AT5G67030	ZEP	1.8	up	Zeaxanthin epoxidase, chloroplastic
AT5G03760	CSLA9	0.3	down	Glucomannan 4-beta-mannosyltransferase 9
AT5G45340	CYP707A3	0.3	down	Abscisic acid 8′-hydroxylase 3
AT2G26150	HSFA2	0.2	down	Heat stress transcription factor A-2

## Supplementary Materials

Supplementary materials can be found at https://www.mdpi.com/1422-0067/21/9/3121/s1.

## Abbreviations

ABA	abscisic acid
BR	brassinosteroids
DEGs	differentially expressed genes
FDR	false discovery rate
FPKM	Fragments per Kilobase per Million Mapped Fragments
GFP	green fluorescent protein
Hsf	heat shock factor
Hsp	heat shock protein
MAS	mannopine synthase
MeJA	methyl jasmonate
NAA	a-naphthaleneacetic acid
qPCR	real-time quantitative PCR
