# Supplementary material for: Wheat Heat Shock Factor TaHsfA6f Increases ABA Levels and Enhances Tolerance to Multiple Abiotic Stresses in Transgenic Plants
Source: Int J Mol Sci. 2020 Apr 28;21(9):3121. doi: 10.3390/ijms21093121 (PMC7247712; doi:10.3390/ijms21093121)
Supplement: Supplementary file 1 [file ijms-21-03121-s001.pdf]

Supplementary Material

Supplementary Figures and Tables

Figure S1 Sequence analysis of *TaHsfA6f* gene. (A) Gene structure analysis of *TaHsfA6f.* (B) Phylogenetic analysis of TaHsfA6f and its homologous proteins from other plants.

Table S1. Primer sequences used in this study.

Table S2. List of assembled transcripts that were up-regulated or down-regulated (log2 fold change (log2FC) > 1 or <-1, false discovery rate (FDR) < 0.001) in *TaHsfA6f* transgenic *Arabidopsis* plants relative to WT plants.

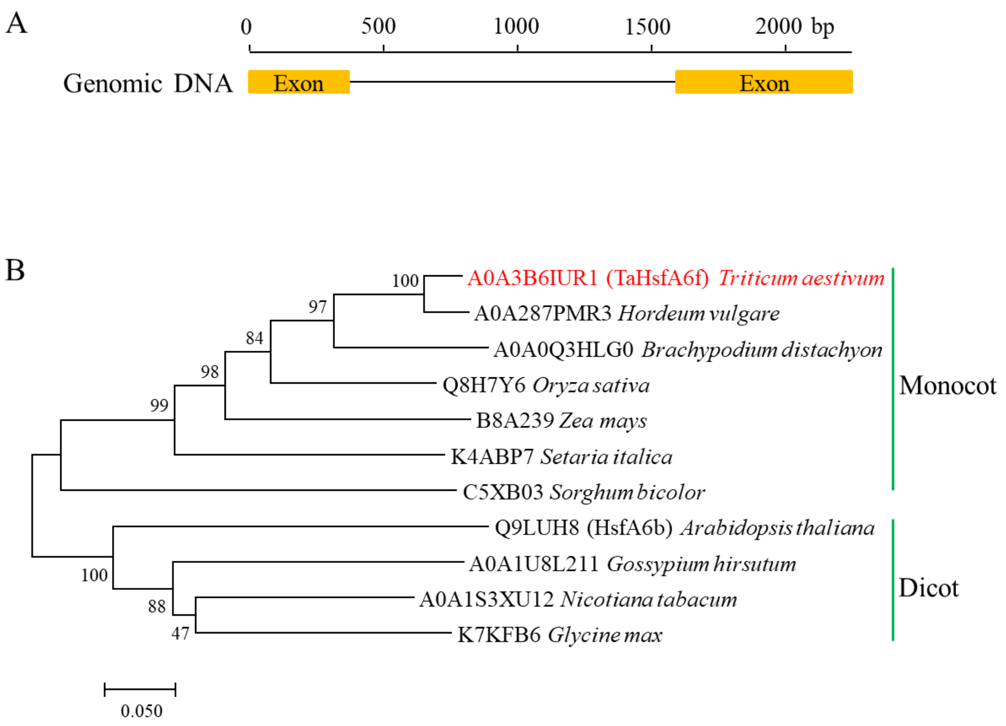

Figure S1 Sequence analysis of *TaHsfA6f* gene. (A) Gene structure analysis of *TaHsfA6f*. (B) Phylogenetic analysis of TaHsfA6f and its homologous proteins from other plants.

Table S1. Primer sequences used in this study.

Reactions	Primer name	Sequence information (5′ to 3′)	Enzyme site
Genomic	TaHsfA6f-GF	GCTGGTTGTTTGCTTGCT	
	TaHsfA6f-GR	CGCTCTTACTTAGATGACCCA	
cDNA	TaHsfA6f-cF	GCTGGTTGTTTGCTTGCT	
	TaHsfA6f-cR	CGCTCTTACTTAGATGACCCA	
qRT-PCR	TaHsfA6f-qF	CTTGAGGACGCTTATTCGAAC	
	TaHsfA6f-qR	CATGGCTAAATTCTCCAGCTC	
	β-actin-F	GGAATCCATGAGACCACCTAC	
	β-actin-R	GACCCAGACAACTCGCAAC	
Subcellular localization	TaHsfA6f-SF	GGGGACGAGCTCGGTACCATGGACCGGGTGCTGCT	*Kpn* I
	TaHsfA6f-SR	CATGGTGTCGACTCTAGACGCGTCGACATATCTAG	*Xba* I
Transgenic *Arabidopsis*	TaHsfA6f-TF	ATACACCAAATCGACTCTAGAATGGACCGGGTGCTGCT	*Xba* I
	TaHsfA6f-TR	CGATCGGGGAAATTCGAGCTCCTACGCGTCGACATATCT	*Sac* I
RNA-seq analysis	LTP3-F	TGGCTCCATGTGCAACCTAT	
	LTP3-R	GGACTGGATGCATCTGCAAG	
	HsfA2-F	GGAGGAACAATGTTTGGAGG	
	HsfA2-R	GACCGCAACAAGTAGATGTG	
	ZEP-F	ATGCCATCGATGCTTGACTG	
	ZEP-R	ATCACAATGCGCATTCCAGG	
	CYP707A3-F	ATGGATTTCTCCGGTTTGTTTC	
	CYP707A3-R	CTATGGTTTTCGTTCCAAGG	
	Actin2-F	GCTCCTCTTAACCCAAAGGC	
	Actin2-R	CACACCATCACCAGAATCCAGC	

Table S2. List of assembled transcripts that were up-regulated or down-regulated (log2 fold change (log2FC) > 1 or <-1, false discovery rate (FDR) < 0.001) in *TaHsfA6f* transgenic *Arabidopsis* plants relative to WT plants.

Gene ID	log2FC	P-value	FDR	Profile	Gene name
AT2G06845	9.87	9.66E-17	5.46E-15	up	
AT4G10250	9.40	7.01E-20	5.64E-18	up	HSP22.0
AT1G60470	9.16	1.49E-14	6.57E-13	up	GOLS4
AT4G27670	8.89	7.58E-34	2.00E-31	up	HSP25.3
AT2G05510	8.61	1.11E-12	3.94E-11	up	
AT4G11650	8.54	1.23E-12	4.37E-11	up	OSM34
AT3G09640	8.07	2.09E-11	6.34E-10	up	APX2
AT5G43300	7.31	5.13E-09	1.04E-07	up	GDPD3
AT5G07330	7.28	7.57E-09	1.50E-07	up	
AT2G39310	7.23	8.81E-09	1.74E-07	up	JAL22
AT1G52560	7.22	5.61E-12	1.85E-10	up	HSP26.5
AT5G41590	7.18	1.45E-08	2.77E-07	up	
AT5G59720	6.39	1.54E-229	9.72E-226	up	HSP18.1
AT5G15250	6.34	1.69E-28	3.05E-26	up	FTSH6
AT1G73260	5.84	6.32E-47	3.75E-44	up	KTI1
AT3G09260	5.74	1.24E-19	9.65E-18	up	BGLU23
AT2G19900	5.67	5.92E-05	0.000511	up	NADP-ME1
AT4G33720	5.58	7.46E-05	0.000629	up	
AT1G53540	5.53	2.73E-43	1.29E-40	up	HSP17.6C
AT1G72660	5.29	2.59E-06	3.13E-05	up	DRG2
AT3G49110	5.03	1.16E-99	2.44E-96	up	PER33
AT4G25200	4.98	9.29E-10	2.17E-08	up	HSP23.6
AT3G13784	4.81	3.20E-07	4.63E-06	up	CWINV5
AT2G43580	4.64	7.24E-46	3.93E-43	up	
AT5G44430	4.61	4.50E-88	7.12E-85	up	PDF1.2C
AT3G09450	4.57	4.22E-08	7.32E-07	up	
AT4G31615	4.53	0.000105	0.000853	up	REM2
AT1G60970	4.29	1.32E-05	0.000135	up	
AT5G59310	4.25	1.19E-30	2.55E-28	up	LTP4
AT2G23110	4.20	2.95E-06	3.51E-05	up	
AT1G62760	4.10	3.06E-05	0.000281	up	PMEI10
AT2G26020	4.08	9.62E-178	4.57E-174	up	PDF1.2B
AT3G12500	4.06	2.51E-93	4.33E-90	up	CHI-B
AT2G43590	4.04	7.52E-103	1.79E-99	up	
AT5G44420	4.02	8.52E-255	1.62E-250	up	PDF1.2A
AT1G30220	3.97	1.00E-15	5.11E-14	up	INT2
AT2G05440	3.84	5.58E-108	1.51E-104	up	ATGRP9
AT3G49620	3.74	3.22E-82	4.71E-79	up	DIN11

AT3G14680	3.64	2.94E-19	2.15E-17	up	CYP72A14
AT5G12020	3.57	5.53E-16	2.90E-14	up	HSP17.6
AT2G26010	3.43	2.38E-253	2.26E-249	up	PDF1.3
AT4G16260	3.43	6.78E-130	2.15E-126	up	
AT3G44550	3.34	8.59E-11	2.38E-09	up	FAR5
AT4G08770	3.31	2.14E-17	1.31E-15	up	PER37
AT4G21320	3.28	8.50E-32	2.04E-29	up	HSA32
AT4G22050	3.19	0.000105	0.000854	up	
AT5G12030	3.16	6.18E-23	6.75E-21	up	HSP17.7
AT2G47180	3.14	2.66E-97	5.05E-94	up	GOLS1
AT4G21323	2.97	3.66E-09	7.62E-08	up	
AT3G28740	2.93	1.63E-15	8.06E-14	up	CYP81D11
AT4G01390	2.88	1.90E-23	2.17E-21	up	
AT2G36750	2.83	1.68E-05	0.000166	up	UGT73C1
AT4G23493	2.74	3.50E-13	1.32E-11	up	
AT5G19110	2.70	9.14E-06	9.74E-05	up	
AT2G29500	2.69	4.18E-48	2.56E-45	up	HSP17.6B
AT5G38780	2.69	2.99E-06	3.55E-05	up	
AT5G47610	2.64	3.62E-31	8.29E-29	up	ATL79
AT1G19510	2.62	1.33E-09	2.99E-08	up	RL5
AT5G22300	2.51	5.49E-35	1.58E-32	up	NIT4
AT1G34060	2.49	1.29E-14	5.75E-13	up	TAR4
AT1G07260	2.46	4.24E-23	4.68E-21	up	UGT71C3
AT1G66060	2.46	2.65E-09	5.66E-08	up	
AT1G48000	2.42	3.97E-09	8.22E-08	up	MYB112
AT2G01008	2.41	3.52E-07	5.05E-06	up	
AT5G10520	2.37	6.85E-19	4.79E-17	up	RBK1
AT4G25100	2.36	3.19E-14	1.36E-12	up	FSD1
AT2G18050	2.36	2.36E-63	2.24E-60	up	HIS1-3
AT4G31330	2.35	2.08E-11	6.33E-10	up	
AT3G19270	2.34	2.26E-07	3.38E-06	up	CYP707A4
AT3G04720	2.34	1.65E-66	1.65E-63	up	HEL
AT1G47510	2.33	4.62E-08	7.95E-07	up	IP5P11
AT5G61160	2.33	1.75E-44	8.72E-42	up	ACT
AT5G23020	2.32	2.06E-31	4.83E-29	up	MAM3
AT1G01070	2.29	2.41E-07	3.58E-06	up	
AT1G09500	2.29	3.07E-05	0.000281	up	
AT4G23680	2.26	1.01E-22	1.07E-20	up	
AT3G23550	2.23	2.93E-57	2.53E-54	up	DTX18
AT5G19875	2.21	5.65E-27	8.93E-25	up	
AT1G09480	2.20	9.13E-13	3.29E-11	up	
AT1G02820	2.18	1.12E-05	0.000116	up	LEA3
AT5G59320	2.15	4.89E-34	1.35E-31	up	LTP3
AT1G33950	2.15	2.23E-06	2.72E-05	up	IAN7
AT5G37670	2.12	6.85E-22	6.78E-20	up	HSP15.7

AT4G33550	2.09	1.35E-07	2.11E-06	up	
AT1G02230	2.05	6.95E-06	7.63E-05	up	NAC004
AT2G15020	2.04	8.82E-17	5.00E-15	up	
AT1G62290	2.02	4.70E-35	1.37E-32	up	APA2
AT4G06746	2.00	1.33E-23	1.58E-21	up	RAP2-9
AT1G66700	1.99	1.28E-16	7.08E-15	up	PXMT1
AT1G73330	1.97	8.62E-10	2.02E-08	up	ATDR4
AT1G64360	1.95	4.27E-37	1.45E-34	up	
AT5G15830	1.93	3.63E-06	4.26E-05	up	AtbZIP3
AT1G06160	1.92	2.42E-31	5.61E-29	up	ERF094
AT3G27400	1.91	4.11E-20	3.46E-18	up	
AT3G05955	1.91	1.23E-08	2.39E-07	up	
AT4G15280	1.88	1.20E-06	1.54E-05	up	UGT71B5
AT2G46830	1.87	3.54E-25	4.91E-23	up	CCA1
AT5G41761	1.86	2.67E-27	4.29E-25	up	
AT2G22800	1.84	1.11E-13	4.46E-12	up	HAT9
AT4G08290	1.81	1.46E-24	1.87E-22	up	
AT2G05914	1.81	3.93E-06	4.57E-05	up	
AT3G61880	1.81	2.65E-12	9.08E-11	up	CYP78A9
AT4G12500	1.80	0.000117	0.000942	up	
AT4G37990	1.80	2.25E-05	0.000215	up	CAD8
AT4G19645	1.79	1.99E-13	7.78E-12	up	
AT5G03640	1.79	1.22E-07	1.93E-06	up	
AT4G35900	1.77	3.77E-06	4.39E-05	up	FD
AT2G44480	1.76	4.65E-11	1.36E-09	up	BGLU17
AT5G45080	1.76	1.46E-06	1.85E-05	up	PP2A6
AT3G44970	1.73	1.95E-06	2.41E-05	up	
AT3G61990	1.72	1.47E-17	9.07E-16	up	OMTF3
AT1G66830	1.72	4.08E-08	7.08E-07	up	
AT4G20820	1.66	1.11E-05	0.000116	up	
AT4G02280	1.66	7.99E-08	1.31E-06	up	SUS3
AT4G16000	1.64	2.53E-12	8.69E-11	up	
AT5G12110	1.61	1.36E-19	1.06E-17	up	
AT2G02930	1.61	8.05E-06	8.71E-05	up	GSTF3
AT5G09220	1.61	6.20E-57	5.12E-54	up	AAP2
AT1G16030	1.60	6.50E-24	8.01E-22	up	HSP70-5
AT2G37130	1.59	5.63E-25	7.70E-23	up	PER21
AT5G16570	1.59	3.90E-19	2.80E-17	up	GLN1-4
AT2G32120	1.59	6.60E-10	1.57E-08	up	HSP70-8
AT1G49470	1.59	3.84E-17	2.28E-15	up	
AT5G13170	1.59	0.000111	0.0009	up	SWEET15
AT4G26150	1.57	3.37E-07	4.84E-06	up	GATA22
AT5G58770	1.57	2.54E-19	1.90E-17	up	
AT4G25000	1.57	4.08E-22	4.19E-20	up	AMY1

AT2G34960	1.55	4.62E-05	0.000405	up	CAT5
AT1G15125	1.54	4.25E-37	1.45E-34	up	
AT4G04450	1.53	1.75E-05	0.000172	up	WRKY42
AT3G22740	1.53	5.76E-22	5.76E-20	up	HMT3
AT2G46720	1.53	1.55E-09	3.47E-08	up	HIC
AT4G12470	1.52	4.77E-23	5.23E-21	up	AZI1
AT1G03020	1.50	2.52E-06	3.05E-05	up	GRXS1
AT4G34250	1.50	4.57E-06	5.22E-05	up	KCS16
AT3G17070	1.49	1.12E-14	5.03E-13	up	PER29
AT5G14940	1.49	5.93E-11	1.68E-09	up	NPF5.8
AT1G70300	1.49	8.38E-18	5.27E-16	up	POT6
AT3G15356	1.49	1.09E-38	4.22E-36	up	LEC
AT4G12480	1.48	4.41E-16	2.36E-14	up	EARLI1
AT5G55970	1.48	2.46E-15	1.19E-13	up	
AT2G37870	1.47	1.06E-05	0.000111	up	
AT5G58390	1.46	5.80E-17	3.38E-15	up	PER67
AT3G01970	1.46	1.23E-10	3.31E-09	up	WRKY45
AT2G41800	1.46	1.09E-06	1.41E-05	up	
AT5G04150	1.46	4.11E-20	3.46E-18	up	BHLH101
AT2G26650	1.44	1.55E-12	5.46E-11	up	AKT1
AT5G47590	1.43	2.53E-06	3.06E-05	up	
AT1G21100	1.42	1.05E-09	2.41E-08	up	IGMT1
AT2G04160	1.42	3.77E-21	3.46E-19	up	AIR3
AT1G16310	1.41	2.64E-05	0.000248	up	MTP10
AT2G38530	1.41	1.84E-08	3.43E-07	up	LTP2
AT4G08300	1.40	2.29E-17	1.40E-15	up	
AT5G23980	1.39	4.41E-19	3.16E-17	up	FRO4
AT5G15500	1.39	5.06E-11	1.45E-09	up	
AT1G47960	1.39	8.99E-22	8.80E-20	up	C/VIF1
AT4G29190	1.38	5.97E-69	6.67E-66	up	
AT2G22240	1.38	1.36E-16	7.54E-15	up	IPS2
AT3G55130	1.37	5.02E-38	1.80E-35	up	ABCG19
AT1G06180	1.37	1.44E-08	2.74E-07	up	ATMYB13
AT5G19880	1.37	9.74E-06	0.000103	up	PER58
AT5G39610	1.37	2.13E-11	6.46E-10	up	NAC92
AT3G48740	1.36	1.24E-48	8.19E-46	up	SWEET11
AT1G56600	1.35	1.62E-18	1.09E-16	up	GOLS2
AT5G02020	1.34	7.65E-08	1.26E-06	up	SIS
AT1G66725	1.34	1.65E-07	2.55E-06	up	MIR163
AT2G17710	1.34	1.91E-10	4.97E-09	up	
AT5G11100	1.33	5.78E-07	7.89E-06	up	SYTD
AT4G21230	1.33	1.45E-07	2.27E-06	up	CRK27
AT1G76690	1.32	2.02E-12	7.01E-11	up	OPR2
AT3G56240	1.31	3.41E-43	1.58E-40	up	CCH
AT4G19170	1.31	2.78E-42	1.20E-39	up	CCD4

AT5G55930	1.30	1.73E-17	1.06E-15	up	OPT1
AT3G16530	1.30	2.50E-26	3.71E-24	up	
AT2G15480	1.29	7.01E-14	2.88E-12	up	UGT73B5
AT5G23660	1.29	1.25E-29	2.51E-27	up	SWEET12
AT1G58190	1.29	3.59E-07	5.14E-06	up	AtRLP9
AT4G32940	1.29	2.22E-22	2.31E-20	up	GAMMA-VPE
AT3G50970	1.28	1.31E-10	3.50E-09	up	XERO2
AT4G27300	1.28	1.56E-25	2.23E-23	up	SD11
AT3G12580	1.28	1.05E-25	1.52E-23	up	MED37C
AT5G25475	1.27	4.33E-07	6.08E-06	up	
AT1G74650	1.27	5.82E-09	1.17E-07	up	ATY13
AT3G29035	1.27	5.71E-11	1.63E-09	up	NAC59
AT1G25277	1.27	5.36E-10	1.30E-08	up	
AT1G67980	1.27	9.51E-10	2.20E-08	up	CCOAMT
AT4G11290	1.26	1.60E-12	5.61E-11	up	PER39
AT1G07610	1.26	3.51E-11	1.04E-09	up	MT1C
AT5G59780	1.26	3.10E-19	2.25E-17	up	MYB59
AT5G04950	1.26	6.32E-12	2.06E-10	up	NAS1
AT1G07430	1.26	4.22E-11	1.24E-09	up	AIP1
AT5G54585	1.25	1.14E-09	2.61E-08	up	
AT1G78460	1.25	6.28E-34	1.68E-31	up	
AT3G32980	1.24	2.05E-07	3.11E-06	up	PER32
AT5G59700	1.24	1.79E-10	4.67E-09	up	
AT3G53720	1.24	7.47E-13	2.70E-11	up	CHX20
AT1G30190	1.23	4.64E-05	0.000407	up	
AT1G12200	1.23	3.12E-13	1.20E-11	up	
AT1G43650	1.22	1.09E-05	0.000114	up	
AT1G62180	1.22	5.00E-15	2.35E-13	up	APR2
AT2G04170	1.22	1.24E-07	1.95E-06	up	
AT1G21460	1.22	3.06E-10	7.77E-09	up	SWEET1
AT4G19370	1.22	3.54E-09	7.38E-08	up	
AT5G49730	1.21	2.98E-22	3.09E-20	up	FRO6
AT1G30250	1.20	4.55E-42	1.92E-39	up	
AT4G29110	1.20	2.87E-28	5.04E-26	up	
AT1G25530	1.20	1.67E-05	0.000165	up	
AT1G80570	1.19	1.29E-06	1.65E-05	up	FBL14
AT2G32870	1.17	3.36E-24	4.28E-22	up	
AT4G27450	1.17	4.66E-21	4.23E-19	up	
AT1G12010	1.17	1.87E-06	2.32E-05	up	
AT5G20960	1.17	1.80E-09	3.98E-08	up	AAO1
AT3G14990	1.16	1.81E-48	1.15E-45	up	DJ1A
AT4G21870	1.16	1.52E-06	1.92E-05	up	HSP15.4
AT1G80440	1.16	4.91E-39	1.94E-36	up	
AT4G25480	1.15	1.75E-26	2.66E-24	up	DREB1A

AT3G48390	1.15	4.87E-05	0.000425	up	
AT1G19250	1.15	4.29E-05	0.000379	up	FMO1
AT3G62150	1.15	7.09E-28	1.21E-25	up	ABCB21
AT4G37520	1.15	2.40E-16	1.31E-14	up	PER50
AT1G08810	1.14	1.39E-06	1.76E-05	up	MYB60
AT4G14480	1.14	3.48E-05	0.000314	up	BLUS1
AT2G16720	1.14	1.15E-09	2.63E-08	up	MYB7
AT3G12320	1.13	5.06E-11	1.45E-09	up	LNK3
AT1G60730	1.13	6.31E-25	8.55E-23	up	
AT3G55646	1.13	6.08E-07	8.27E-06	up	
AT1G33030	1.13	2.57E-05	0.000242	up	
AT4G24670	1.12	9.28E-05	0.000766	up	TAR2
AT4G25470	1.11	9.05E-12	2.90E-10	up	DREB1C
AT3G03270	1.10	5.13E-10	1.25E-08	up	
AT3G62930	1.10	3.16E-05	0.000288	up	GRXS6
AT1G67870	1.09	2.06E-27	3.35E-25	up	
AT3G29034	1.09	9.14E-08	1.48E-06	up	
AT5G07100	1.09	1.52E-14	6.72E-13	up	WRKY26
AT1G21000	1.09	5.40E-22	5.45E-20	up	
AT2G24260	1.08	1.78E-06	2.23E-05	up	BHLH66
AT5G66400	1.08	1.91E-10	4.97E-09	up	RAB18
AT5G49740	1.08	3.16E-37	1.11E-34	up	FRO7
AT4G01450	1.07	1.56E-14	6.87E-13	up	
AT1G21310	1.07	7.33E-20	5.88E-18	up	EXT3
AT1G64370	1.07	1.25E-48	8.19E-46	up	
AT3G57020	1.07	1.54E-07	2.40E-06	up	SSL9
AT2G36970	1.06	2.41E-17	1.46E-15	up	UGT86A1
AT3G29670	1.06	6.66E-07	8.99E-06	up	PMAT2
AT1G71960	1.05	2.24E-08	4.12E-07	up	ABCG25
AT2G36800	1.05	5.38E-06	6.03E-05	up	UGT73C5
AT5G58650	1.05	0.000111	0.000903	up	PSY1
AT1G35260	1.05	2.24E-05	0.000214	up	MLP165
AT3G14280	1.05	4.99E-11	1.44E-09	up	
AT1G24400	1.04	2.70E-08	4.88E-07	up	LHT2
AT1G52870	1.03	3.39E-35	1.04E-32	up	
ATCG00220	1.03	2.90E-06	3.47E-05	up	PSBM
AT3G10450	1.03	2.44E-09	5.24E-08	up	SCPL7
AT1G16400	1.03	3.05E-07	4.44E-06	up	CYP79F2
AT1G77885	1.03	1.07E-05	0.000112	up	
AT5G56870	1.03	3.51E-14	1.49E-12	up	BGAL4
AT4G24480	1.02	8.15E-06	8.80E-05	up	
AT5G61590	1.02	4.77E-38	1.74E-35	up	ERF107
AT1G80830	1.01	3.17E-10	8.00E-09	up	NRAMP1
AT1G68740	1.01	8.65E-06	9.29E-05	up	PHO1-H1
AT1G67810	1.01	1.03E-16	5.75E-15	up	SUFE2

AT1G70420	1.01	7.47E-25	9.92E-23	up	
AT3G50740	1.01	9.48E-15	4.30E-13	up	UGT72E1
AT2G37460	1.01	4.63E-13	1.72E-11	up	
AT1G62810	1.00	2.01E-15	9.83E-14	up	
AT4G27410	1.00	4.65E-11	1.36E-09	up	RD26
AT3G62720	-1.00	5.07E-08	8.66E-07	down	XXT1
AT3G21190	-1.00	3.17E-09	6.68E-08	down	
AT5G11740	-1.00	5.09E-26	7.44E-24	down	AGP15
AT1G67470	-1.01	2.83E-10	7.20E-09	down	
AT2G22500	-1.01	2.66E-12	9.08E-11	down	PUMP5
AT5G58620	-1.01	1.36E-10	3.63E-09	down	
AT4G23220	-1.01	4.52E-19	3.22E-17	down	CRK14
AT3G43670	-1.01	9.95E-07	1.30E-05	down	
AT2G44040	-1.02	1.09E-05	0.000114	down	DAPB1
AT1G67880	-1.02	3.95E-15	1.87E-13	down	
AT1G65010	-1.02	8.35E-16	4.31E-14	down	
AT5G59650	-1.02	8.77E-05	0.000729	down	
AT2G23810	-1.02	9.38E-13	3.37E-11	down	TET8
AT4G02100	-1.02	1.25E-07	1.97E-06	down	
AT2G41100	-1.02	3.77E-12	1.26E-10	down	CML12
AT5G07030	-1.02	7.24E-10	1.71E-08	down	
AT4G34390	-1.02	1.55E-18	1.05E-16	down	XLG2
AT2G13820	-1.02	3.05E-05	0.00028	down	
AT2G06050	-1.03	2.84E-17	1.71E-15	down	OPR3
AT4G14750	-1.03	4.73E-07	6.60E-06	down	IQD19
AT5G47380	-1.03	3.38E-05	0.000306	down	
AT1G76040	-1.03	4.42E-13	1.66E-11	down	CPK29
AT1G70740	-1.03	9.12E-12	2.92E-10	down	
AT5G08050	-1.03	1.99E-11	6.08E-10	down	
AT4G12420	-1.03	4.53E-13	1.69E-11	down	SKU5
AT5G56980	-1.03	2.31E-08	4.23E-07	down	
AT3G57780	-1.03	1.67E-06	2.10E-05	down	
AT3G56370	-1.04	1.03E-09	2.37E-08	down	IRK
AT4G13250	-1.04	2.22E-23	2.51E-21	down	NYC1
AT1G29660	-1.04	2.53E-20	2.16E-18	down	
AT3G55430	-1.04	4.61E-16	2.45E-14	down	
AT3G54810	-1.04	7.33E-16	3.80E-14	down	GATA8
AT5G25460	-1.05	3.86E-13	1.46E-11	down	
AT5G18660	-1.05	1.00E-08	1.96E-07	down	DVR
AT1G02390	-1.05	1.74E-05	0.000172	down	GPAT2
AT1G69900	-1.05	1.33E-06	1.70E-05	down	
AT5G63180	-1.05	1.02E-15	5.19E-14	down	
AT1G56660	-1.06	1.41E-05	0.000143	down	
AT3G23805	-1.06	7.69E-06	8.36E-05	down	RALFL24

AT1G72970	-1.06	3.08E-05	0.000282	down	HTH
AT2G33570	-1.06	1.19E-05	0.000123	down	GALS1
AT2G43550	-1.06	6.62E-17	3.81E-15	down	ATTI6
AT5G13060	-1.06	5.66E-05	0.00049	down	ABAP1
AT1G58848	-1.06	9.33E-05	0.00077	down	RF9
AT5G41900	-1.07	1.78E-05	0.000175	down	BDG2
AT1G10340	-1.07	1.51E-13	5.95E-12	down	
AT1G73805	-1.07	2.89E-19	2.13E-17	down	SARD1
AT1G18250	-1.07	7.71E-06	8.38E-05	down	ATLP-1
AT1G06830	-1.07	2.38E-05	0.000226	down	GRXS11
AT5G62570	-1.07	1.06E-13	4.26E-12	down	
AT3G44860	-1.07	1.99E-16	1.09E-14	down	FAMT
AT1G56650	-1.08	1.18E-08	2.30E-07	down	MYB75
AT4G34900	-1.08	1.57E-13	6.15E-12	down	XDH2
AT5G44582	-1.08	9.95E-05	0.000814	down	
AT5G45950	-1.08	1.40E-07	2.20E-06	down	
AT1G01680	-1.08	1.23E-05	0.000127	down	PUB54
AT3G53760	-1.08	1.88E-07	2.87E-06	down	GCP4
AT2G04495	-1.09	1.38E-09	3.11E-08	down	
AT2G39330	-1.09	5.01E-13	1.84E-11	down	JAL23
AT1G58290	-1.09	2.18E-25	3.06E-23	down	HEMA1
AT5G51560	-1.09	1.10E-06	1.43E-05	down	
AT4G24380	-1.09	2.92E-08	5.21E-07	down	
AT5G26920	-1.09	3.09E-13	1.18E-11	down	CBP60G
AT2G25270	-1.09	4.54E-05	0.000399	down	
AT3G28220	-1.09	1.92E-16	1.05E-14	down	
AT1G13250	-1.10	1.51E-13	5.95E-12	down	GATL3
AT2G18680	-1.10	5.12E-05	0.000446	down	
AT5G20740	-1.10	1.91E-05	0.000186	down	
AT5G15870	-1.10	1.39E-11	4.31E-10	down	
AT3G57460	-1.11	6.67E-13	2.43E-11	down	
AT5G11550	-1.11	6.34E-10	1.52E-08	down	
AT3G13437	-1.11	1.27E-09	2.88E-08	down	
AT2G17880	-1.11	5.67E-09	1.15E-07	down	
AT2G40140	-1.11	6.95E-15	3.21E-13	down	CZF1
AT1G58420	-1.11	2.59E-06	3.13E-05	down	
AT5G47230	-1.11	4.94E-15	2.33E-13	down	ERF5
AT4G27280	-1.11	2.25E-05	0.000215	down	
AT1G17620	-1.11	5.94E-22	5.91E-20	down	
AT1G30520	-1.12	5.82E-09	1.17E-07	down	AAE14
AT1G76160	-1.12	1.00E-27	1.70E-25	down	sks5
AT1G68330	-1.12	9.28E-05	0.000766	down	
AT1G19300	-1.13	4.35E-20	3.61E-18	down	GATL1
AT2G41540	-1.13	4.75E-07	6.61E-06	down	GPDHC1
AT1G70090	-1.13	1.30E-14	5.79E-13	down	GATL9

AT3G06035	-1.13	1.84E-09	4.04E-08	down	
AT1G70370	-1.14	3.12E-29	6.05E-27	down	PGL3
AT3G20470	-1.14	6.48E-08	1.08E-06	down	GRP5
AT3G50280	-1.14	4.79E-07	6.65E-06	down	
AT1G03870	-1.14	3.62E-17	2.16E-15	down	FLA9
AT1G12080	-1.14	6.62E-05	0.000565	down	
AT4G23150	-1.15	4.60E-17	2.71E-15	down	CRK7
AT4G37800	-1.15	4.77E-38	1.74E-35	down	XTH7
AT3G08720	-1.15	9.89E-13	3.55E-11	down	ATPK2
AT1G24170	-1.15	1.45E-18	9.85E-17	down	GATL8
AT3G51450	-1.16	7.37E-25	9.85E-23	down	SSL7
AT2G28950	-1.17	1.05E-14	4.76E-13	down	EXPA6
AT1G64610	-1.17	1.42E-13	5.63E-12	down	
AT3G18710	-1.17	6.58E-08	1.09E-06	down	PUB29
AT2G17280	-1.17	8.34E-06	8.98E-05	down	PGM
AT3G10720	-1.17	2.10E-21	2.01E-19	down	PME25
AT5G08020	-1.17	4.11E-06	4.77E-05	down	RPA1B
AT4G30850	-1.18	2.95E-05	0.000272	down	HHP2
AT5G05190	-1.18	2.78E-17	1.68E-15	down	EDR4
AT4G08870	-1.19	2.52E-25	3.52E-23	down	ARGAH2
AT4G23180	-1.19	3.68E-31	8.33E-29	down	CRK10
AT3G56090	-1.19	6.97E-11	1.95E-09	down	FER3
AT3G07320	-1.19	1.35E-08	2.58E-07	down	
AT1G19180	-1.20	2.44E-18	1.62E-16	down	TIFY10A
AT2G13610	-1.20	3.10E-08	5.49E-07	down	ABCG5
AT4G39670	-1.20	1.07E-10	2.91E-09	down	
AT4G04510	-1.21	9.96E-05	0.000815	down	CRK38
AT2G38750	-1.22	1.43E-08	2.74E-07	down	ANN4
AT5G22545	-1.22	3.72E-06	4.34E-05	down	
AT2G15390	-1.22	2.15E-19	1.65E-17	down	FUT4
AT1G27770	-1.22	1.18E-27	1.99E-25	down	ACA1
AT2G47060	-1.23	5.12E-34	1.39E-31	down	
AT4G18640	-1.23	1.59E-05	0.000158	down	MDIS2
AT3G57230	-1.23	8.65E-22	8.51E-20	down	AGL16
AT3G59080	-1.23	2.98E-13	1.15E-11	down	
AT2G36200	-1.23	5.29E-06	5.95E-05	down	
AT1G30280	-1.23	9.50E-05	0.000782	down	
AT4G13340	-1.24	3.15E-32	7.77E-30	down	LRX3
AT2G34150	-1.24	8.63E-08	1.40E-06	down	SCAR1
AT5G22580	-1.24	3.06E-21	2.86E-19	down	
AT4G35320	-1.24	3.33E-10	8.34E-09	down	
AT5G52760	-1.24	4.25E-14	1.78E-12	down	HIPP14
AT5G66070	-1.24	4.92E-17	2.89E-15	down	
AT1G09932	-1.25	1.70E-15	8.39E-14	down	

AT2G44500	-1.25	7.78E-23	8.42E-21	down	
AT2G40820	-1.25	3.35E-10	8.38E-09	down	
AT1G16420	-1.26	1.59E-05	0.000158	down	AMC8
AT1G02400	-1.26	5.78E-06	6.43E-05	down	GA2OX6
AT4G23200	-1.26	2.80E-08	5.02E-07	down	CRK12
AT4G01950	-1.26	6.53E-13	2.38E-11	down	ATGPAT3
AT1G04160	-1.26	2.70E-05	0.000253	down	XI-B
AT5G23400	-1.26	7.41E-05	0.000625	down	
AT1G17750	-1.27	2.81E-05	0.000261	down	PEPR2
AT1G60060	-1.27	4.86E-06	5.53E-05	down	
AT2G37640	-1.27	6.35E-17	3.67E-15	down	EXPA3
AT3G06140	-1.27	9.64E-06	0.000102	down	LUL4
AT5G03120	-1.27	4.15E-23	4.61E-21	down	
AT1G12845	-1.27	4.88E-14	2.04E-12	down	
AT3G06770	-1.28	3.16E-15	1.51E-13	down	
AT1G19380	-1.28	4.30E-16	2.31E-14	down	
AT1G25250	-1.28	2.50E-07	3.70E-06	down	AtIDD16
AT5G39860	-1.28	2.38E-05	0.000226	down	PRE1
AT2G26190	-1.28	8.38E-23	8.99E-21	down	IQM4
AT5G42860	-1.28	3.27E-14	1.39E-12	down	
AT3G17120	-1.28	6.48E-19	4.55E-17	down	
AT5G49520	-1.29	7.19E-09	1.43E-07	down	WRKY48
AT2G46780	-1.29	8.03E-05	0.000672	down	
AT1G43910	-1.29	4.26E-07	5.99E-06	down	
AT3G49530	-1.29	4.61E-24	5.75E-22	down	NAC062
AT2G33580	-1.30	5.69E-21	5.09E-19	down	LYK5
AT5G26220	-1.31	3.45E-12	1.17E-10	down	GGCT2
AT3G22790	-1.31	8.54E-10	2.00E-08	down	NET1A
AT3G15540	-1.31	1.01E-10	2.76E-09	down	IAA19
AT2G32990	-1.31	4.02E-09	8.30E-08	down	AtGH9B8
AT5G13840	-1.32	2.36E-05	0.000225	down	FZR3
AT1G63750	-1.33	6.64E-18	4.22E-16	down	
AT4G18440	-1.33	1.11E-72	1.32E-69	down	
AT5G01900	-1.33	2.59E-05	0.000244	down	WRKY62
AT3G03850	-1.33	2.85E-06	3.41E-05	down	
AT1G72940	-1.33	3.85E-05	0.000344	down	
AT5G44130	-1.33	4.06E-15	1.92E-13	down	FLA13
AT5G47500	-1.33	8.91E-07	1.17E-05	down	PME68
AT3G25180	-1.34	6.51E-06	7.17E-05	down	CYP82G1
AT4G21070	-1.34	2.79E-05	0.000259	down	BRCA1
AT2G40300	-1.34	4.21E-05	0.000372	down	FER4
AT2G18690	-1.34	6.45E-19	4.55E-17	down	
AT4G22010	-1.35	5.90E-10	1.42E-08	down	sks4
AT3G48360	-1.35	1.82E-23	2.10E-21	down	BT2
AT1G33760	-1.35	2.16E-10	5.58E-09	down	ERF022

AT5G64040	-1.35	1.65E-08	3.10E-07	down	PSAN
AT3G48630	-1.35	8.33E-07	1.10E-05	down	
AT2G23130	-1.35	2.58E-12	8.87E-11	down	AGP17
AT2G16750	-1.36	2.73E-14	1.18E-12	down	
AT1G72920	-1.36	8.31E-06	8.95E-05	down	
AT1G04680	-1.36	5.09E-20	4.16E-18	down	
AT3G23450	-1.36	5.06E-12	1.68E-10	down	
AT4G24010	-1.36	6.44E-07	8.74E-06	down	CSLG1
AT1G13650	-1.37	1.19E-07	1.89E-06	down	
AT5G48900	-1.37	5.08E-12	1.68E-10	down	
AT5G60800	-1.37	7.90E-17	4.51E-15	down	
AT2G33330	-1.37	1.83E-08	3.43E-07	down	CRRSP11
AT5G26030	-1.37	3.66E-26	5.39E-24	down	FC1
AT4G36240	-1.38	1.37E-08	2.62E-07	down	GATA7
AT1G09950	-1.38	1.38E-05	0.00014	down	RAS1
AT2G41170	-1.38	3.68E-05	0.00033	down	
AT4G12720	-1.39	1.14E-22	1.20E-20	down	AtNUDT7
AT3G12110	-1.39	3.60E-07	5.14E-06	down	ACT11
AT1G73080	-1.40	7.80E-23	8.42E-21	down	PEPR1
AT5G02890	-1.40	2.01E-08	3.73E-07	down	
AT2G45470	-1.40	1.33E-07	2.10E-06	down	FLA8
AT2G17040	-1.40	5.15E-24	6.40E-22	down	anac036
AT2G32930	-1.40	1.72E-15	8.50E-14	down	ZFN2
AT4G14770	-1.41	4.97E-06	5.64E-05	down	TCX2
AT1G50010	-1.41	1.42E-06	1.80E-05	down	TUBA4
AT1G79680	-1.41	4.70E-13	1.74E-11	down	WAKL10
AT2G01180	-1.42	9.11E-21	8.04E-19	down	ATPAP1
AT1G26560	-1.42	2.37E-20	2.04E-18	down	BGLU40
AT4G39830	-1.42	1.24E-16	6.90E-15	down	
AT4G23490	-1.43	1.44E-06	1.82E-05	down	
AT5G09800	-1.43	2.33E-05	0.000222	down	PUB28
ATCG01180	-1.43	9.13E-06	9.73E-05	down	RRN23S.2
AT1G42990	-1.43	2.22E-28	3.97E-26	down	BZIP60
AT3G01550	-1.43	7.20E-10	1.71E-08	down	PPT2
AT5G52882	-1.43	2.87E-15	1.38E-13	down	
AT4G38950	-1.44	8.83E-12	2.84E-10	down	KIN7F
AT5G55730	-1.44	2.53E-10	6.44E-09	down	FLA1
AT3G12700	-1.45	7.42E-08	1.22E-06	down	
AT1G21120	-1.45	2.60E-08	4.71E-07	down	
AT1G60950	-1.46	4.60E-36	1.48E-33	down	FD2
AT2G34060	-1.46	2.43E-05	0.00023	down	PER19
AT2G25297	-1.46	0.000113	0.000914	down	
AT3G23810	-1.46	1.49E-36	4.95E-34	down	SAHH2
AT1G65390	-1.48	6.09E-09	1.22E-07	down	PP2A5

AT4G31000	-1.48	1.08E-14	4.88E-13	down	CBP60F
AT5G66210	-1.49	2.75E-33	7.05E-31	down	CPK28
AT3G28180	-1.50	6.17E-46	3.45E-43	down	CSLC4
AT4G08390	-1.50	8.86E-21	7.86E-19	down	APXS
AT3G30720	-1.50	1.27E-17	7.88E-16	down	QQS
AT1G24140	-1.50	2.87E-23	3.21E-21	down	3MMP
AT5G65390	-1.50	8.61E-12	2.77E-10	down	AGP7
AT4G37450	-1.51	2.26E-20	1.95E-18	down	AGP18
AT1G72240	-1.51	2.20E-09	4.77E-08	down	
AT3G09940	-1.51	4.86E-13	1.79E-11	down	MDAR3
AT1G59910	-1.51	1.44E-28	2.66E-26	down	FH7
AT1G22330	-1.52	6.38E-12	2.08E-10	down	
AT4G26660	-1.52	2.75E-05	0.000256	down	
AT4G14090	-1.52	7.01E-06	7.68E-05	down	UGT75C1
AT3G52370	-1.53	1.63E-10	4.27E-09	down	FLA15
AT1G49010	-1.53	8.81E-17	5.00E-15	down	
AT2G14890	-1.53	1.17E-24	1.52E-22	down	AGP9
AT2G35190	-1.53	1.03E-11	3.27E-10	down	NPSN11
AT4G29780	-1.53	5.40E-06	6.04E-05	down	
AT2G46400	-1.54	3.57E-05	0.000321	down	WRKY46
AT1G29980	-1.54	2.60E-13	1.01E-11	down	
AT1G64640	-1.54	2.97E-30	6.13E-28	down	ENODL8
AT4G21850	-1.54	4.60E-18	2.97E-16	down	MSRB9
AT4G32460	-1.55	9.18E-16	4.71E-14	down	
AT5G25190	-1.55	2.91E-19	2.13E-17	down	ERF003
AT3G29030	-1.55	1.64E-35	5.11E-33	down	EXPA5
AT1G04520	-1.55	8.97E-09	1.77E-07	down	CRRSP3
AT3G45970	-1.56	1.44E-29	2.84E-27	down	EXLA1
AT5G65280	-1.56	0.000111	0.000903	down	GCL1
AT3G19970	-1.56	6.75E-14	2.78E-12	down	
AT1G05675	-1.56	1.22E-10	3.28E-09	down	UGT74E1
AT2G39650	-1.56	3.19E-16	1.73E-14	down	
AT2G38470	-1.56	2.68E-28	4.76E-26	down	WRKY33
AT3G03820	-1.57	3.66E-06	4.28E-05	down	
AT3G08860	-1.57	6.31E-11	1.78E-09	down	PYD4
AT3G46090	-1.58	2.14E-17	1.31E-15	down	ZAT7
AT1G09080	-1.58	4.89E-14	2.04E-12	down	MED37B
AT4G34150	-1.58	1.15E-34	3.26E-32	down	
AT5G41460	-1.59	5.06E-14	2.10E-12	down	
AT3G12610	-1.59	4.15E-31	9.16E-29	down	DRT100
AT5G07990	-1.59	5.62E-06	6.26E-05	down	CYP75B1
AT3G50800	-1.59	3.55E-14	1.50E-12	down	
AT1G64390	-1.59	3.56E-24	4.47E-22	down	AtGH9C2
AT5G22460	-1.60	4.94E-29	9.38E-27	down	
AT5G67070	-1.61	4.53E-29	8.69E-27	down	RALFL34

AT1G66465	-1.61	1.28E-11	4.01E-10	down	
AT3G57530	-1.61	3.96E-35	1.17E-32	down	CPK32
AT5G17700	-1.61	1.77E-07	2.72E-06	down	DTX25
AT5G39320	-1.61	1.48E-17	9.12E-16	down	UGD4
AT3G04220	-1.62	2.71E-07	3.98E-06	down	
AT5G51465	-1.62	6.69E-15	3.10E-13	down	
AT5G05270	-1.62	7.92E-07	1.05E-05	down	CHI3
AT5G45280	-1.62	1.07E-23	1.30E-21	down	PAE11
AT3G11640	-1.62	7.47E-05	0.000629	down	
AT1G02205	-1.63	1.87E-09	4.10E-08	down	CER1
AT5G58120	-1.63	1.21E-29	2.45E-27	down	
AT4G36950	-1.63	1.59E-06	2.00E-05	down	MAPKKK21
AT2G32140	-1.64	1.46E-18	9.93E-17	down	
AT1G76620	-1.64	7.59E-11	2.12E-09	down	
AT3G50120	-1.65	1.45E-10	3.83E-09	down	
AT1G80840	-1.65	3.06E-07	4.45E-06	down	WRKY40
AT5G17170	-1.65	3.79E-25	5.21E-23	down	ENH1
AT2G34620	-1.66	2.73E-20	2.31E-18	down	
AT5G49170	-1.67	1.17E-06	1.51E-05	down	
AT3G02840	-1.67	1.92E-15	9.44E-14	down	
AT1G60270	-1.67	1.64E-08	3.09E-07	down	BGLU6
AT5G24105	-1.67	3.77E-06	4.39E-05	down	AGP41
AT3G46490	-1.67	2.90E-09	6.16E-08	down	
AT2G29110	-1.68	7.50E-14	3.05E-12	down	ATGLR2.8
AT3G10660	-1.69	4.13E-06	4.78E-05	down	CPK2
AT3G21950	-1.69	1.52E-05	0.000152	down	
AT3G51470	-1.70	1.00E-08	1.96E-07	down	
AT4G20830	-1.70	2.46E-30	5.14E-28	down	
AT5G65920	-1.70	5.80E-18	3.69E-16	down	PUB31
AT5G44050	-1.70	1.54E-05	0.000154	down	DTX28
AT5G22520	-1.70	5.25E-14	2.18E-12	down	
AT5G03760	-1.71	2.48E-26	3.70E-24	down	CSLA9
AT3G03030	-1.71	1.65E-08	3.10E-07	down	
AT2G25460	-1.71	3.23E-07	4.67E-06	down	
AT1G30040	-1.72	1.13E-07	1.80E-06	down	GA2OX2
AT3G55980	-1.72	1.19E-11	3.75E-10	down	SZF1
AT1G57630	-1.73	3.00E-15	1.43E-13	down	
AT5G25260	-1.74	9.92E-09	1.95E-07	down	FLOT2
AT1G29690	-1.74	2.28E-33	5.93E-31	down	CAD1
AT5G09470	-1.74	4.39E-09	9.04E-08	down	PUMP6
AT5G13140	-1.76	2.94E-21	2.77E-19	down	
AT5G42380	-1.77	1.83E-20	1.61E-18	down	CML37
AT5G67450	-1.77	1.06E-19	8.41E-18	down	AZF1
AT5G02490	-1.77	5.00E-40	2.02E-37	down	MED37D

AT1G17420	-1.79	3.12E-34	8.72E-32	down	LOX3
AT4G23190	-1.79	6.68E-27	1.05E-24	down	CRK11
AT3G23250	-1.79	1.88E-06	2.33E-05	down	MYB15
AT3G16860	-1.80	7.29E-14	2.99E-12	down	COBL8
AT3G27940	-1.80	2.12E-23	2.41E-21	down	LBD26
AT4G37240	-1.80	6.06E-10	1.46E-08	down	
AT4G23210	-1.80	3.98E-12	1.33E-10	down	CRK13
AT3G48100	-1.80	1.82E-27	2.98E-25	down	ARR5
AT5G35740	-1.81	8.59E-08	1.40E-06	down	
AT3G28580	-1.83	2.74E-15	1.32E-13	down	
AT2G24850	-1.83	7.80E-46	4.12E-43	down	TAT3
AT3G43960	-1.84	4.51E-08	7.78E-07	down	RDL3
AT1G16130	-1.85	2.19E-09	4.75E-08	down	WAKL2
AT5G42040	-1.86	1.23E-05	0.000127	down	RPN12B
AT2G24600	-1.87	1.05E-35	3.33E-33	down	
AT5G16200	-1.87	1.58E-12	5.56E-11	down	
AT5G45340	-1.87	2.83E-27	4.51E-25	down	CYP707A3
AT4G14365	-1.87	7.40E-47	4.26E-44	down	XBAT34
AT2G04780	-1.88	3.68E-21	3.40E-19	down	FLA7
AT2G37025	-1.88	1.61E-05	0.00016	down	TRFL8
AT3G16240	-1.88	6.32E-08	1.06E-06	down	TIP2-1
AT1G28480	-1.89	1.64E-23	1.90E-21	down	GRXC9
AT5G48850	-1.90	4.02E-14	1.69E-12	down	SDI1
AT1G23205	-1.90	2.57E-06	3.10E-05	down	
AT2G20142	-1.90	1.08E-26	1.68E-24	down	
AT5G22380	-1.91	1.06E-06	1.37E-05	down	NAC090
AT2G01422	-1.91	4.38E-22	4.47E-20	down	
AT2G22170	-1.93	6.52E-57	5.16E-54	down	PLAT2
AT4G17470	-1.93	0.000123	0.000987	down	
AT5G15780	-1.94	1.47E-05	0.000148	down	
AT1G24070	-1.97	1.06E-14	4.78E-13	down	CSLA10
AT2G47780	-1.97	1.97E-05	0.000192	down	
AT2G21540	-1.97	1.46E-12	5.15E-11	down	SFH3
AT2G34600	-2.00	7.29E-07	9.76E-06	down	TIFY
AT3G42800	-2.00	8.96E-06	9.59E-05	down	
AT1G61800	-2.01	1.24E-27	2.06E-25	down	GPT2
AT5G22940	-2.02	2.29E-07	3.42E-06	down	F8H
AT1G11850	-2.02	9.78E-05	0.000802	down	
AT3G10310	-2.02	1.22E-05	0.000125	down	
AT2G06850	-2.03	1.29E-68	1.36E-65	down	XTH4
AT2G27402	-2.03	4.45E-16	2.37E-14	down	
AT5G08000	-2.05	1.71E-09	3.81E-08	down	E13L3
AT3G14870	-2.06	8.85E-11	2.44E-09	down	
AT5G24770	-2.06	2.85E-40	1.18E-37	down	VSP2
AT1G01560	-2.07	2.04E-06	2.51E-05	down	MPK11

AT4G08950	-2.07	4.05E-32	9.85E-30	down	EXO
AT1G42980	-2.07	5.18E-08	8.84E-07	down	FH12
AT1G17380	-2.08	1.31E-23	1.56E-21	down	TIFY11A
AT1G61890	-2.09	1.42E-45	7.28E-43	down	DTX37
AT4G23810	-2.11	1.60E-49	1.13E-46	down	WRKY53
AT4G01080	-2.12	1.90E-32	4.74E-30	down	TBL26
AT5G28237	-2.12	6.40E-06	7.06E-05	down	
AT1G20310	-2.12	2.32E-08	4.25E-07	down	
AT1G75780	-2.14	1.60E-19	1.23E-17	down	TUBB1
AT3G50930	-2.16	1.56E-15	7.81E-14	down	HSR4
AT4G38560	-2.17	3.59E-36	1.18E-33	down	
AT1G61120	-2.18	6.08E-16	3.17E-14	down	GES
AT1G50745	-2.18	2.15E-05	0.000207	down	
AT3G21150	-2.18	2.34E-12	8.09E-11	down	BBX32
AT3G06880	-2.18	8.51E-19	5.88E-17	down	
AT5G61440	-2.19	2.84E-13	1.10E-11	down	ACHT5
AT1G53520	-2.20	4.50E-05	0.000396	down	FAP3
AT3G44350	-2.22	2.46E-19	1.85E-17	down	anac061
AT5G52050	-2.22	7.01E-31	1.53E-28	down	DTX50
AT4G02330	-2.25	3.56E-35	1.07E-32	down	PME41
AT3G54400	-2.27	2.38E-21	2.27E-19	down	
AT1G09750	-2.28	2.22E-50	1.62E-47	down	AED3
AT3G15720	-2.31	5.34E-15	2.50E-13	down	
AT1G17830	-2.31	1.54E-09	3.46E-08	down	
AT1G77640	-2.33	2.16E-07	3.27E-06	down	ERF013
AT4G13410	-2.35	1.81E-05	0.000177	down	CSLA15
AT2G26150	-2.35	6.40E-10	1.53E-08	down	HSFA2
AT1G11545	-2.39	7.07E-28	1.21E-25	down	XTH8
AT4G14370	-2.40	8.37E-18	5.27E-16	down	
AT3G48520	-2.40	1.27E-08	2.46E-07	down	CYP94B3
AT1G52750	-2.41	1.21E-05	0.000125	down	
AT1G51620	-2.41	8.62E-09	1.70E-07	down	
AT2G23000	-2.41	3.39E-10	8.47E-09	down	SCPL10
AT4G38400	-2.44	5.98E-09	1.20E-07	down	EXLA2
AT4G28780	-2.47	4.55E-22	4.62E-20	down	
AT4G22880	-2.49	1.24E-16	6.90E-15	down	LDOX
AT3G02410	-2.49	9.34E-06	9.92E-05	down	ICMEL2
AT5G38120	-2.50	1.44E-07	2.25E-06	down	4CLL8
AT1G52830	-2.50	3.29E-09	6.90E-08	down	IAA6
AT5G41740	-2.52	1.63E-58	1.47E-55	down	
AT4G12730	-2.53	1.62E-07	2.51E-06	down	FLA2
AT4G15160	-2.53	1.50E-05	0.00015	down	
AT5G08640	-2.54	2.95E-08	5.26E-07	down	FLS1
AT3G06355	-2.57	3.01E-29	5.89E-27	down	

AT1G11740	-2.57	1.24E-05	0.000128	down	
AT1G21910	-2.60	1.90E-30	4.02E-28	down	ERF012
AT5G47850	-2.62	1.01E-42	4.44E-40	down	CCR4
AT3G01830	-2.63	1.74E-18	1.17E-16	down	CML40
AT4G04460	-2.64	1.35E-05	0.000137	down	APA3
AT4G08040	-2.64	1.98E-06	2.44E-05	down	ACS11
AT3G23730	-2.66	1.55E-11	4.78E-10	down	XTH16
AT3G29250	-2.69	0.000119	0.000955	down	SDR4
AT1G66090	-2.71	9.73E-18	6.08E-16	down	
AT5G41750	-2.71	4.13E-79	5.60E-76	down	
AT3G16670	-2.72	6.22E-11	1.76E-09	down	
AT5G01100	-2.75	4.70E-44	2.29E-41	down	
AT2G20150	-2.75	2.42E-05	0.000229	down	
AT1G54020	-2.75	1.74E-11	5.33E-10	down	
AT2G44840	-2.82	3.75E-38	1.42E-35	down	ERF13
AT5G50915	-2.84	4.37E-20	3.61E-18	down	BHLH137
AT2G21650	-2.85	3.46E-22	3.57E-20	down	RL2
AT4G11320	-2.85	4.13E-43	1.87E-40	down	RDL5
AT1G43000	-2.87	7.57E-07	1.01E-05	down	
AT5G17220	-2.87	3.42E-21	3.18E-19	down	GSTF12
AT1G30370	-2.92	1.51E-07	2.35E-06	down	
AT1G72520	-2.94	1.01E-76	1.27E-73	down	LOX4
AT5G37950	-2.95	4.34E-06	4.98E-05	down	
AT2G10940	-2.95	5.94E-06	6.59E-05	down	
AT2G24610	-2.99	2.74E-05	0.000256	down	ATCNGC14
AT5G36925	-3.03	1.04E-09	2.38E-08	down	
AT4G21830	-3.03	1.13E-07	1.79E-06	down	MSRB7
AT1G50750	-3.10	3.29E-18	2.15E-16	down	
AT2G36885	-3.22	3.02E-05	0.000278	down	
AT1G62440	-3.30	7.00E-06	7.67E-05	down	LRX2
AT1G35140	-3.30	3.92E-31	8.77E-29	down	EXL1
AT5G13930	-3.33	1.22E-52	9.30E-50	down	CHS
AT1G65890	-3.38	3.76E-19	2.71E-17	down	AAE12
AT4G33790	-3.43	9.73E-17	5.48E-15	down	FAR3
AT3G53010	-3.60	4.82E-07	6.70E-06	down	
AT2G38240	-3.64	2.14E-05	0.000207	down	ANS
AT1G10550	-3.65	1.77E-11	5.41E-10	down	XTH33
AT5G42800	-3.74	1.19E-24	1.54E-22	down	DFRA
AT5G35525	-3.75	5.05E-05	0.00044	down	PCR3
AT1G60590	-3.78	1.10E-07	1.76E-06	down	
AT1G51460	-3.91	9.54E-05	0.000784	down	ABCG13
AT1G73325	-3.93	1.20E-19	9.35E-18	down	
AT1G10640	-4.00	5.12E-06	5.78E-05	down	
AT5G54060	-4.02	1.06E-12	3.77E-11	down	A3G2XYLT
AT4G16590	-4.25	1.06E-29	2.17E-27	down	ATCSLA01

AT3G29590	-4.31	2.89E-08	5.18E-07	down	5MAT
AT1G06360	-4.39	5.98E-05	0.000516	down	
AT4G15210	-4.52	8.33E-156	3.16E-152	down	BAM5
AT1G75945	-4.83	6.28E-17	3.63E-15	down	
AT3G60920	-5.31	6.61E-25	8.90E-23	down	
AT4G11911	-5.72	9.34E-06	9.92E-05	down	
AT1G65450	-6.45	9.63E-07	1.26E-05	down	
AT3G44006	-7.43	2.73E-09	5.82E-08	down	
